# ErbB4 in Laminated Brain Structures: A Neurodevelopmental Approach to Schizophrenia

**DOI:** 10.3389/fncel.2015.00472

**Published:** 2015-12-18

**Authors:** Carlos G. Perez-Garcia

**Affiliations:** Molecular Neurobiology Laboratory, The Salk Institute, La JollaCA, USA

**Keywords:** NRG1, ErbB, cortex, cerebellum, hippocampus, neurodevelopmental disorders, schizophrenia

## Abstract

The susceptibility genes for schizophrenia Neuregulin-1 (NRG1) and ErbB4 have critical functions during brain development and in the adult. Alterations in the ErbB4 signaling pathway cause a variety of neurodevelopmental defects including deficiencies in neuronal migration, synaptic plasticity, and myelination. I have used the *ErbB4^-/-^ HER4^heart^* KO mice to study the neurodevelopmental insults associated to deficiencies in the NRG1-ErbB4 signaling pathway and their potential implication with brain disorders such as schizophrenia, a chronic psychiatric disease affecting 1% of the population worldwide. ErbB4 deletion results in an array of neurodevelopmental deficits that are consistent with a schizophrenic model. First, similar defects appear in multiple brain structures, from the cortex to the cerebellum. Second, these defects affect multiple aspects of brain development, from deficits in neuronal migration to impairments in excitatory/inhibitory systems, including reductions in brain volume, cortical and cerebellar heterotopias, alterations in number and distribution of specific subpopulations of interneurons, deficiencies in the astrocytic and oligodendrocytic lineages, and additional insults in major brain structures. This suggests that alterations in specific neurodevelopmental genes that play similar functions in multiple neuroanatomical structures might account for some of the symptomatology observed in schizophrenic patients, such as defects in cognition. ErbB4 mutation uncovers flaws in brain development that are compatible with a neurodevelopmental model of schizophrenia, and it establishes a comprehensive model to study the basis of the disorder before symptoms are detected in the adult.

## Introduction

Brain development is a tightly regulated process wherein multiple signaling pathways cooperate to establish an intricate neuronal network that connects different structures in the central nervous system (CNS). It has been shown that developmental insults are strongly linked to many neurological disorders (Harrison, 1999; Walsh et al., 2008; Muraki and Tanigaki, 2015). Whether this is due to cumulative defects or to major neurodevelopmental disruptions often remains unclear.

Schizophrenia is a complex neurological disorder that affects multiple neuroanatomical structures and has a strong developmental component (Harrison, 1999; Walsh et al., 2008; Muraki and Tanigaki, 2015). It is not caused by defects in a single gene, but rather is a multigenic disorder to which many genes contribute. Understanding neurodevelopmental insults that might lead to schizophrenia is a critical step to conceptualize the driving factors that might cause the disease.

Components of the Neuregulin (NRG)/ErbB signaling pathway display a strong genetic association to the etiology of schizophrenia (Harrison, 1999; Walsh et al., 2008; Buonanno, 2010). The NRG family is comprised of four members, including NRG1, which is highly expressed embryonically and early postnatally in the CNS, and decreases with age (Buonanno, 2010). NRG1 binds ErbB receptors, which are receptor tyrosine kinases of the epidermal growth factor (EGF) family, comprising four members (ErbB1-4) (Yarden and Sliwkowski, 2001). ErbB4 is the main receptor for NRG1 in the CNS and is expressed both embryonically and in the adult (Gerecke et al., 2001; Yau et al., 2003). The NRG1-ErbB pathway is critical for the proliferation, differentiation, and glial cell survival (Adlkofer and Lai, 2000). It is important in the induction of expression of receptors for GABA, NMDA, and acetylcholine (Ozaki et al., 1997; Rieff et al., 1999; Liu et al., 2001); for the development of Schwann cells in the peripheral nervous system (PNS; Canoll et al., 1996); for cortical interneuronal migration (Flames et al., 2004; Li et al., 2012); and as a regulator of dendritogenesis in the cerebellum (Rieff and Corfas, 2006).

The genes encoding NRG1, ErbB3, and ErbB4 have been identified as susceptibility genes for schizophrenia (Corfas et al., 2004; Benzel et al., 2007; Law et al., 2007; Walsh et al., 2008; Shi et al., 2009; Buonanno, 2010), and a disruption in the ErbB4 gene has been described in schizophrenic patients (Walsh et al., 2008). Many studies using mouse models of NRG1 and ErbB4 have shown schizophrenia-related behaviors that can be reversed using antipsychotic drugs (Stefansson et al., 2002; Golub et al., 2004; O’Tuathaigh et al., 2007; Chen et al., 2008, 2010; Mei and Xiong, 2008; Shamir et al., 2012).

In the present study, I focus on finding a cause-effect relationship between neurodevelopmental insults associated with genetic deletion of ErbB4 and the development of schizophrenia in the adult. Since the conventional complete *ErbB4* knock-out (KO) mice die embryonically due to a profound failure in cardiac myocyte differentiation (Gassmann et al., 1995), I have used the *ErbB4^-/-^ HER^heart^* mice as a model. In the *ErbB4^-/-^ HER^heart^* mice, the heart defects were rescued by expressing human ErbB4 (HER4) under a cardiac-specific myosin promoter. Thus, the *ErbB4^-/-^ HER^heart^* mice reach adulthood with a functional wild-type (WT) heart but maintaining the same set of defects in the CNS and PNS as observed in the complete conventional ErbB4 KO mice (Tidcombe et al., 2003).

The *ErbB4^-/-^ HER^heart^* mice have been extensively used in functional and behavioral studies of schizophrenia; however, a neuroanatomical characterization of the mouse model has not been analyzed in detail. I document common neurodevelopmental defects in the main brain structures affected in schizophrenia, such as cortex, hippocampus, or cerebellum. The set of defects include: reductions in volume; cortical and cerebellar heterotopias; deficiencies in astrocyte and oligodendrocyte lineages; decreased myelination; disruption in the migration and distribution of specific subtypes of GABAergic interneurons; and flaws in the development of Bergmann glia and cerebellar neurons. Many of the defects I describe in the *ErbB4^-/-^ HER^heart^* mice have been observed in neuroimaging and post-mortem studies of schizophrenic patients (Guidotti et al., 2000; Volz et al., 2000; Harrison and Weinberger, 2005; Andreasen and Pierson, 2008; Hashimoto et al., 2008; Meyer-Lindenberg, 2010; Muraki and Tanigaki, 2015).

### Materials and Methods

### Mouse Lines

All experiments were approved and conducted following the guidelines of the Institutional Animal Care and Use Committee at the Salk Institute and were in full compliance with the guidelines of the National Institutes of Health for the care and use of laboratory animals. Mice were mated, and the morning of vaginal plug identification was designated as E0.5. The morning were pups are born was designated as P0.5. *ErbB4*^+/+^ *HER^heart^* (WT), *ErbB4*^+/-^ *HER^heart^* and *ErbB4*^-/-^ *HER^heart^* (KO) mice were generated and genotyped by PCR as previously described (Tidcombe et al., 2003).

### Histology

Embryonic brains were dissected and fixed accordingly in PFA 2% (wt/vol) or Bouin’s solution. Postnatal brains were perfused and post-fixed with PFA 2% (wt/vol) or Bouin’s solution. Brains were prepared for paraffin and sectioned at 10 μm. For Nissl staining, sections were stained with 0.5% cresyl violet (wt/vol) and then dehydrated and covered. For immunohistochemistry, paraffin sections were rehydrated and rinsed in Phosphate Buffer Saline (PBS). Antigens were unmasked by boiling in citrate buffer (Vector). Primary antibodies used were: rabbit anti-Calretinin (Swant, 1:1500); rabbit anti-Calbindin (Swant, 1:1500); mouse anti-Reelin (Chemicon, 1:400); rabbit anti-GFAP (Neomarkers, 1:250); rabbit anti-Parvalbumin (1:250, Millipore), mouse anti-PCNA (Neomarkers, 1:250), mouse anti-Neurofilament-L (1:100, Cell Signaling); rabbit anti-MBP (Chemicon, 1:250); rabbit anti-Bhlhb5 (1:1000, gift from Sarah E. Ross, University of Pittsburgh, Pittsburgh, PA, USA), rabbit anti-Tbr1 (1:250, gift from Robert Hevner, Seattle Children’s Research Institute, Seattle, WA, USA), rabbit anti-Parvalbumin (1:250, Chemicon) and rabbit anti-ErbB4 (provided by Cary Lai, Neomarkers, Upstate, Santa Cruz). Primary antibodies were incubated in PBS in a humid chamber overnight at room temperature. After rinsing in saline buffer, sections were prepared for immunofluorescence or diaminobenzidine colorimetric reaction (DAB). For immunofluorescence, sections were incubated in the correspondent Alexa-conjugated fluorochrome (Molecular Probes) for 30 min at room temperature and then they were washed in PBS. Cell nuclei were counterstained using DAPI (Vector). Sections were covered and visualized in a fluorescence microscope. For DAB, sections were washed in PBS, and incubated with the correspondent biotinylated secondary antibody (Vector) for 30 min at room temperature and then were washed in PBS. Sections were incubated with the ABC kit (Vectastain, Vector) for 30 min at room temperature and then were rinsed in PBS. Sections were developed using DAB (Sigma). After dehydration, sections were covered and visualized in the light microscope.

### Cell Counting and Statistics

For quantification, a minimum of four sections per animal was counted, and a minimum of three animals was checked (*N* = 3–12). For measurement and quantification, I used the cell counter and measurement analysis plugins from the FIJI software (NIH, http://rsb.info.nih.gov/ij/). The measured mean values were displayed as percent of the WT, and the variance (±) was calculated using SEM. Statistical significance was determined using un-paired two-tailed *t*-test, *P* < 0.05 (^∗^) were considered statistically significant.

## Results

### Neuroanatomical Defects Associated to the ErbB4 Deletion

**FIGURE 1:**
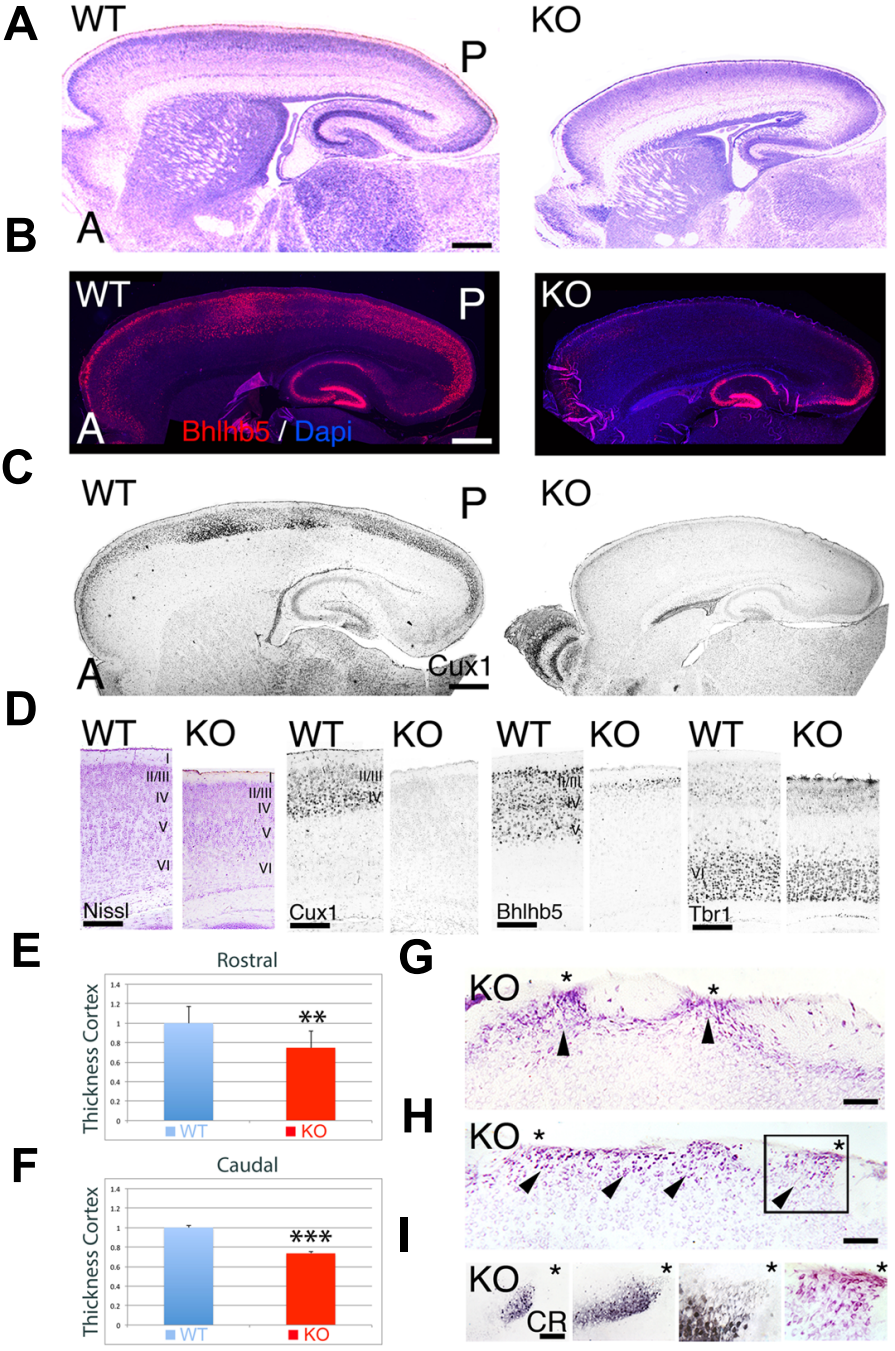
**Neuroanatomical comparison between wild-type (WT) and KO at P7. (A–C)** Sagittal sections of WT and KO stained with Nissl **(A)** or immulabeled for Bhlhb5 **(B)** and Cux1 **(C)**. Upper layer (UL) markers Bhlhb5 and Cux1 are reduced in the cortex of the KO. In **(B)**, DAPI is shown as counterstaining. **(D)** Comparative profile between WT and KO cortical sections stained with Nissl or immunostained for the cortical layer markers Cux1 (II–IV), Bhlhb5 (II–V), and Tbr1 (VI). Cortical lamination is preserved in the KO but ULs are diminished and do not express the mature markers profile characteristic of UL neurons. **(E,F)** Quantitative analysis showing a significant reduction in cortical thickness by 25% at rostral levels (**E**, 0.75 ± 0.061^∗∗^, *p* = 0.0113, *N* = 8) and by 26% at caudal levels (*F*, 0.74 ± 0.009, *p* = 0.0001^∗∗∗^, *N* = 8) in the KO compared to WT. **(G–I)** In the KO, cortical ectopias (arrowheads) are observed in layers II/III and I using Nissl staining **(G,H)** and Calretinin (CR, **I**). Asterisks indicate cortical ectopias reaching the pial surface. A: anterior; I–VI: cortical layers 1–6; P; posterior. Scale bars: **(A–C)** (0.2 mm), **(D)** (125 μm), **(G,H)** (75 μm) and **(I)** (50 μm).

At P7, the *ErbB4^-/-^ HER^heart^* mice (referred as KO) have a reduction along both the rostrocaudal and dorsoventral axes when compared to controls (referred as WT) (**Figure 1A**). Measurements of the cortical thickness indicate that the cortex of KO is significantly reduced by 25% at rostral levels (0.75 ± 0.061^∗∗^, *p* = 0.0113, *N* = 8) and by 26% at caudal levels (0.74 ± 0.009, *p* = 0.0001^∗∗∗^, *N* = 8) when compared to WT (**Figures 1D–F**). Cortical glutamatergic neurons are generated accordingly to a regulated inside-out gradient, where deeper layer neurons (layers 5–6) are generated and migrate earlier than the upper layer (UL) neurons (layers 2–4) (Rakic, 1974). Embryonically, I do not observe any cortical defect associated to the lack of ErbB4. However, when migration is complete, at postnatal (P) day (P7), ULs are reduced in thickness in the KO relative to WT, whereas the thickness of deep layers is preserved (**Figure 1D**). To confirm this, I did immunohistochemistry at P7 using the layer specific markers Cux1 (layers II–IV), Bhlhb5 (layers II–V), and Tbr1 (layer VI). My findings indicate that layer VI appears indistinguishable between KO and WT (**Figure 1D**). Bhlhb5+ cortical neurons are observed in the WT throughout the entire rostrocaudal axis, however, they are almost absent in the KO, with very low expression at the most caudal and rostral poles (**Figures 1B,D**). This result is confirmed using a more restricted UL neuron marker, Cux1, showing that the expression of Cux1 is basically absent from UL neurons, and frequently confined to a thin layer in the rostral and caudal poles (**Figures 1C,D**). In addition, caudal ectopias that contact the pial surface and express neuronal markers are frequently observed in the KO (**Figures 1G–I**). My quantification and layer specific marker analysis, concomitantly with the presence of cortical ectopias, clearly indicates that UL neurons migration and maturation is compromised in the KO mice.

**FIGURE 2:**
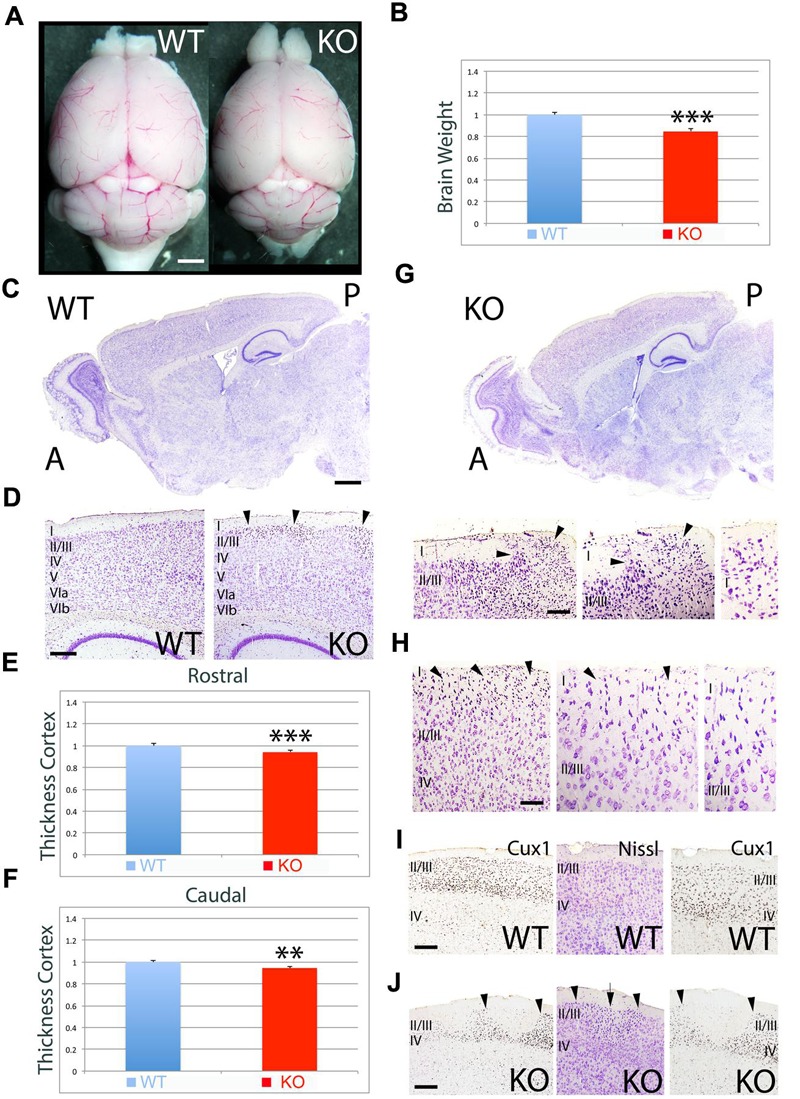
**Neuroanatomical comparison between WT and KO in the adult. (A)** Whole brain comparison between WT and KO. **(B)** Quantitation indicates a significant reduction in brain weight by 15% in the KO (0.85 ± 0.020, *p* = 0.0027^∗∗∗^, *N* = 4). **(C)** Sagittal sections stained with Nissl comparing WT and KO. **(D)** Nissl stained sections comparing the cortical thickness between WT and KO. **(E,F)** Quantitative analysis showing a significant reduction in cortical thickness by 6% at rostral (**E**, 0.94 ± 0.010, *p* = 0.0041^∗∗∗^, *N* = 4) and caudal (*F*, 0.94 ± 0.011, *p* = 0.0101^∗∗^, *N* = 4) levels in the KO compared to WT. **(G,H)** In the KO, cortical ectopias and heterotopias are observed in ULs including layer I. Arrowheads indicate the location of the ectopias in layer I at caudal **(G)** and rostral **(H)** levels. **(I,J)** Immunostaining for the UL marker Cux1 in WT **(I)** and KO **(J)**. Cux1+ UL neurons present an abnormal distribution pattern in the KO **(J)**, where patches of Cux1 negative neurons are observed. In **(J)**, arrowheads mark the limits of the Cux1 negative territory in the ULs where heterotopias (arrow) negative for Cux1 are found. A, anterior; I-VIb, cortical layers 1 to 6b; P, posterior. Scale bars: **(A)** (0.5 mm), **(C)** (0.2 mm), **(D)** (125 μm), **(G)** (75 μm), **(H)** (50 μm), and **(I,J)** (100 μm).

To further investigate the differences observed at P7, I analyzed the adult mice (**Figure 2**). Quantitative measurement of brain weight indicates that there is a significant reduction in brain volume by 15% in the KO versus WT (**Figures 2A,B**, 0.85 ± 0.020, *p* = 0.0027^∗∗∗^, *N* = 4). Overall, the cortical thickness is significantly reduced by 6% at rostral (**Figure 2E**, 0.94 ± 0.010, *p* = 0.0041^∗∗∗^, *N* = 4) and caudal (**Figure 2F**, 0.94 ± 0.011, *p* = 0.0101^∗∗^, *N* = 4) levels in the KO compared to WT (**Figures 2C–F**). Despite the recovery in cell density when compared to P7, the adult cortex still presents severe abnormalities in the ULs such as the presence of ectopias at caudal (**Figure 2G**) and rostral (**Figure 2H**) levels that extent through layer I and contact the pial surface. These ectopias are not observed in WT. Additionally, I observe abnormalities in the expression of UL neuron markers such as Cux1 that frequently coincide with the presence of heterotopias in ULs (**Figures 2I,J**). The neuroanatomical profile presented in this study unveils novel defects associated to the deficiency in ErbB4.

### ErbB4-deficiency in the CNS Alters Astrocytic and Oligodendrocytic Lineages

**FIGURE 3:**
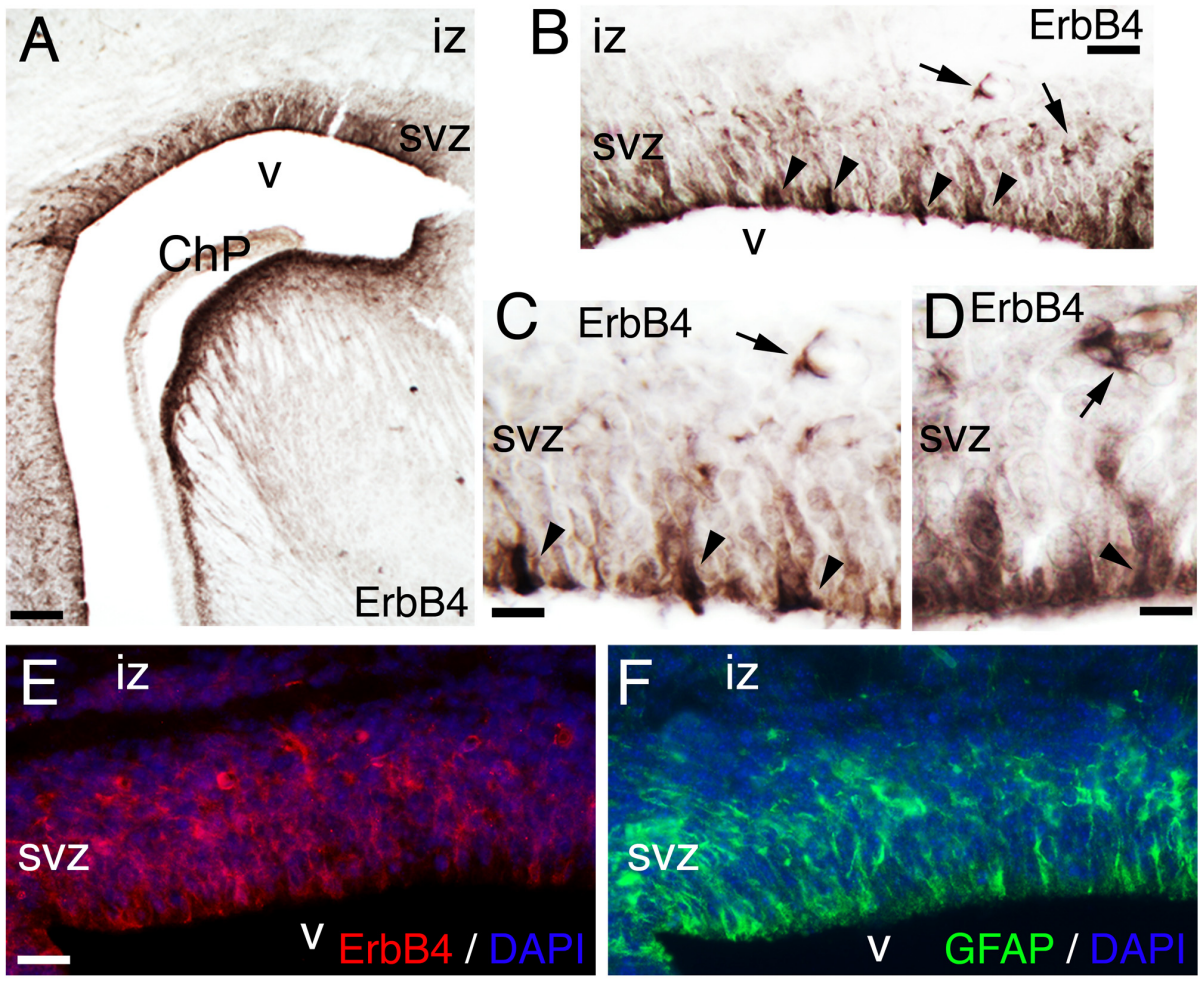
**ErbB4 is expressed in the GFAP positive lineage. (A–F)** E17. **(A–D)** ErbB4 is expressed in the cortical subventricular zone (SVZ, arrows) and in cells with end-feet lining the ventricle (v) and radial morphology (arrowheads). **(C,D)** are high magnification views of **(A,B)**, respectively. **(E,F)** Adjacent sections showing the co-expression pattern of ErbB4 **(E)** and GFAP **(F)** in the SVZ and in radial cells lining the ventricle. DAPI is shown as counterstaining. ChP, choroid plexus; iz, intermediate zone. Scale bars: **(A)** (100 μm), **(B)** (50 μm), **(C,D)** (25 μm), and **(E,F)** (50 μm).

**FIGURE 4:**
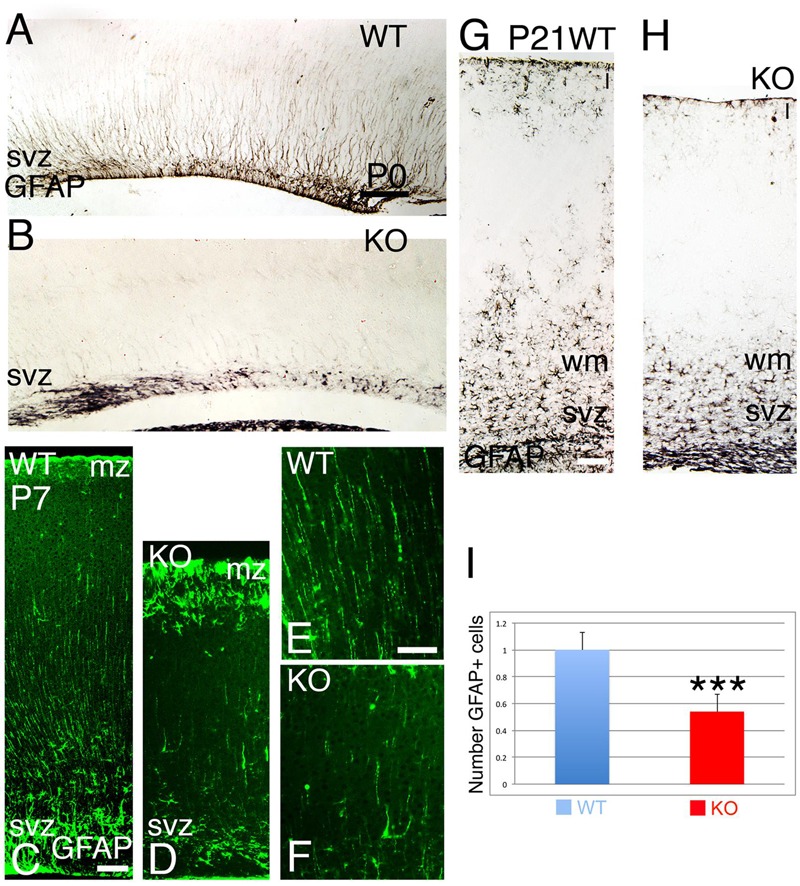
**The development of the astroglia is compromised in the KO. (A,B)** (P0), **(C–F)** (P7), and **(G,H)** (P21) GFAP+ radial fibers are observed in WT from P0 to P7 whereas in KO the GFAP+ fibers are fragmented, misaligned, and reduced in number. At P7, an early astrogenesis is observed in the marginal zone (mz) in the KO with severe reduction in astrocytes in the svz. **(E,F)** are high power views of **(C,D)**, respectively. **(I)**, Quantification analysis indicates that the number of GFAP+ astrocytes in the cortical wall is significantly reduced by 46% (0.54 ± 0.01, *p* = 0.0005^∗∗∗^, *N* = 4) in the KO compared to WT. I, cortical layer 1; wm: white matter. Scale bars: **(A–D)** (100 μm), **(E,F)** (50 μm) and **(G,H)** (125 μm).

At late embryogenesis, ErbB4 begins to be expressed in the astroglial lineage in both neuronal progenitor compartments, SVZ and VZ (**Figure 3**). ErbB4 is expressed in Glial Fibrillary Acidic Protein (GFAP)+ cells attached to the VZ with a glia-like morphology, end-feet lining the ventricle and short processes (**Figure 3**). GFAP is a marker for astrocytes suggesting that the ErbB4 population I am observing belongs to the astrocytic lineage. Within the SVZ, ErbB4+ cells are not attached to the ventricle and co-express GFAP (**Figure 3**). From P0 to P7, GFAP+ astroglia fibers extend radial processes throughout the cortical plate (CP) and attach to the pial surface (**Figures 4A,C,E**). In the KO, GFAP+ astroglia fibers are reduced in number with short processes at P0 (**Figure 4B**) and by P7 the radial fibers are fragmented and misaligned with very short processes (**Figures 4D,F**). Concomitantly in the KO, GFAP+ radial fibers detached from the pial surface earlier than WT and transformed prematurely into astrocytes, whereas at the level of the SVZ, astrocyte number is diminished (**Figures 4C,D**). By P21, the number of GFAP+ astrocytes in the entire cortical wall is significantly reduced by 46% (0.54 ± 0.01, *p* = 0.0005^∗∗∗^, *N* = 4) in the KO compared to WT (**Figures 4G–I**). These data confirm an ErbB4-dependent role in astroglia development.

**FIGURE 5:**
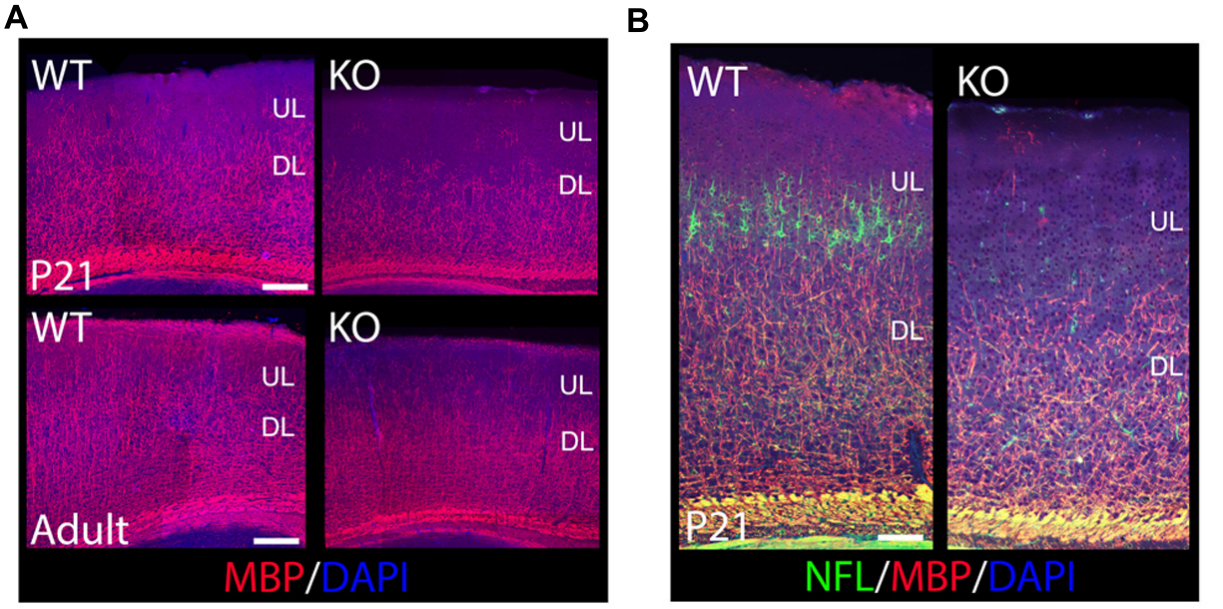
**The oligodendroglia lineage, myelin production, and axonal growth are compromised in the KO. (A)** Shown are cortical sections immunostained for MBP at P21 and in the adult. MBP+ mature oligodendrocytes are reduced in the KO versus WT when labeled with MBP. In the adult, ULs are significantly hypomyelinated in the KO compared to WT. **(B)** Shown are cortical sections co-immunostained for MBP and NFL at P21. Axonal development is reduced in the cortex in the KO, very significant in ULs. DL, deeper layers. Scale bar: **(A)** (100 μm) and **(B)** (125 μm).

To determine if oligodendrocytes are also affected in the KO, I used Myelin Basic Protein (MBP) to label mature oligodendrocytes and myelin at P21 and in the adult (**Figure 5A**). I observe an overall reduction of MBP+ oligodendrocytes in the cortex, with fewer MBP+ fibers extending their processes throughout the CP, which is a crucial step in the initiation of myelination (**Figure 5A**). ULs are significantly hypomyelinated in the adult (**Figure 5A**). My results indicate that ErbB4 is important for oligodendrocyte development and myelin production.

To determine if axonal development was also affected in the KO, I performed double co-localization at P21 by using MBP to label myelinated axons, and Neurofilament-L (NFL) to label the intermediate filaments of mature axons. My data indicates a severe reduction in NFL expression in mature cortical neurons, very significant for ULs (**Figure 5B**), which is consistent with a similar reduction in MBP expression (**Figures 5A,B**). These results indicate a failure of axonal development in cortical neurons in the KO.

### Interneuronal Subpopulations are Selectively Reduced in the KO

ErbB4 is expressed in migrating GABAergic interneurons generated in the subpallium (Yau et al., 2003). ErbB4+ interneurons migrate to the cortex through NRG-dependent chemotrophic mechanisms (Flames et al., 2004; Li et al., 2012). Deficiencies in either NRG1 or ErbB4 have a dramatic impact in the number of GABAergic interneurons (Flames et al., 2004; Li et al., 2012).

To study the behavior of specific cortical subtypes of GABAergic interneurons in the KO model, I selected four major subpopulations of GABAergic interneurons and assessed their migratory routes and final cortical distribution.

**FIGURE 6:**
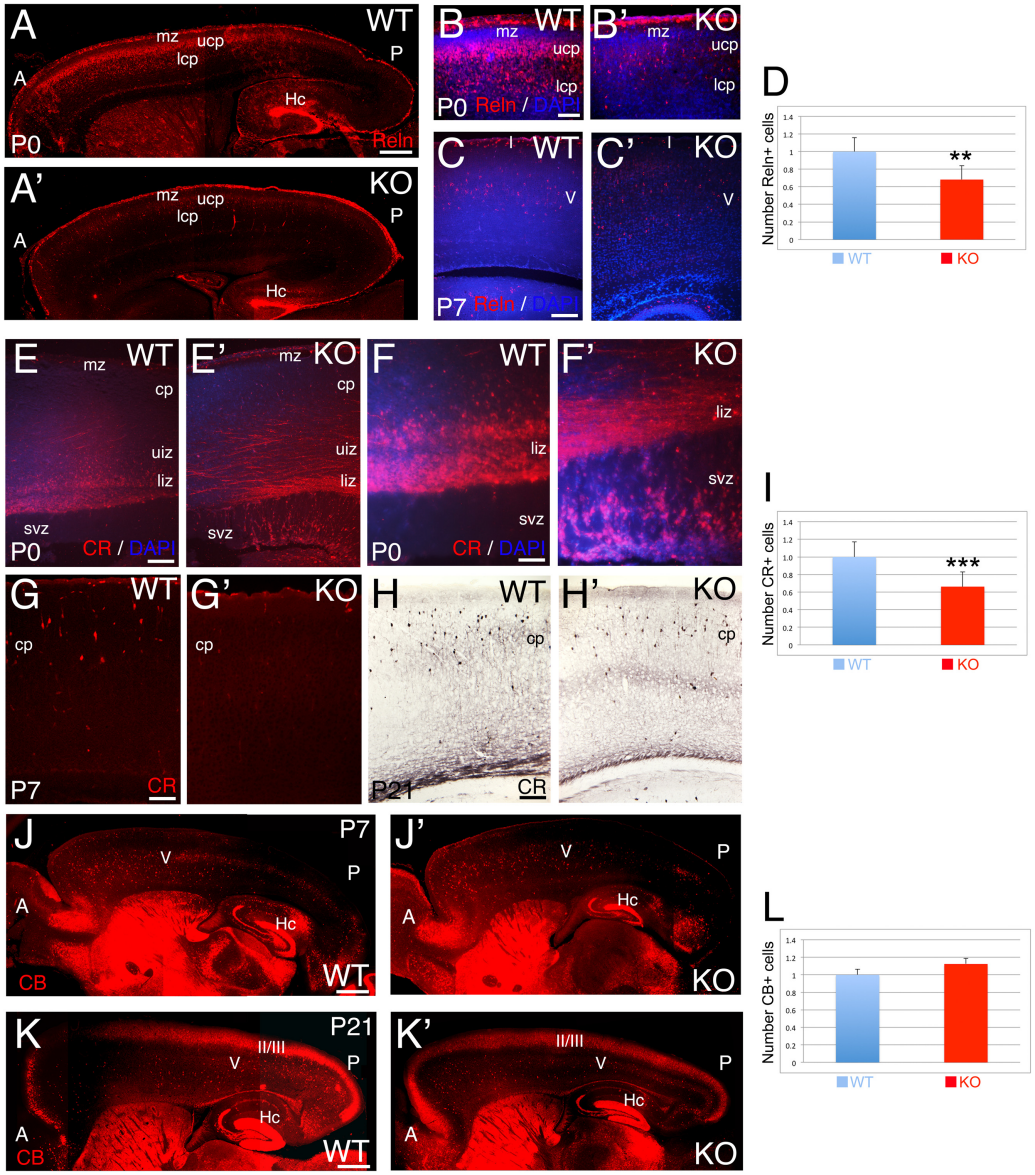
**Selective deficit of GABAergic interneuron subpopulations are observed in KO. (A–C’)** Reelin (Reln)+ interneurons at P0 **(A–B’)** and P7 **(C,C’)** in WT **(A,B,C)** and KO **(A’,B’,C’)**. At P0 WT, Reelin+ interneurons are expressed through a high-anterior to low-posterior gradient at the interface between mz and upper cortical plate (ucp) with radially oriented interneurons also present in the lower cortical plate (lcp). In the KO, the Reelin+ interneuronal gradient is very rudimentary with a severe reduction in the number of Reelin+ interneurons. **(B,B’)** are high power views of **(A,A’)**, respectively. At P7 WT, Reelin+ interneurons are mostly located in layer V in the cortical plate (cp), whereas in KO, the density of Reelin+ interneurons is reduced and they are more dispersed throughout the cortical plate. DAPI is shown as counterstaining. **(D)** At P21, Reelin+ interneurons are significantly reduced by 32% in the cortex of KO compared to WT (0.68 ± 0.07, *p* = 0.0261^∗∗^, *N* = 4). **(E–H’)** Calretinin (CR)+ interneurons at P0 **(E–F’)**, P7 **(G,G’)**, and P21 **(H,H’)** in WT **(E,F,G,H)** and KO **(E’,F’,G’,H’)**. At P0, WT CR+ interneurons are radially oriented in the lower intermediate zone (liz) with subsets of CR+ interneurons already migrating in the upper intermediate zone (uiz). In the KO, a dense plexus of CR+ fibers and tangentially oriented CR+ interneurons are observed in the upper and lower intermediate zones, where the vast majority of radially oriented CR+ interneurons are located in the svz. **(F,F’)** are high power views of **(E,E’)**, respectively. At P7, CR+ interneurons are located in the upper cortical plate in WT and absent in the KO. DAPI is shown as counterstaining. At P21, CR+ interneurons are densely observed in the cortical plate in WT, with a severe reduction in the KO. **(I)** At P21, a statistically significant reduction of CR+ interneurons by 35% is observed in the KO compared to WT (0.66 ± 0.07, *p* = 0.0004^∗∗∗^, *N* = 9). **(J–K’)** Calbindin (CB)+ interneurons at P7 **(J,J’)** and P21 **(K,K’)** in WT **(J,K)** and KO **(J’,K’)**. At P7, CB+ interneurons are mostly located in layer V in both WT and KO, but at posterior levels their number is severely reduced with defects in their distribution pattern. At P21, the majority of CB+ interneurons are located in layers II/III and V. DAPI is shown as counterstaining. L: Quantification of CB+ interneurons indicates no statistically difference between WT and KO at P21 (1.12 ± 0.01, *p* = 0.186, *N* = 8). A, anterior axis; Hc, hippocampus; I, cortical layer 1; II/III: cortical layers 2/3; P, posterior axis; V, cortical layer 5. Scale bars: **(A,A’)** (0.2 mm), **(B,B’)** (50 μm), **(C,C’)** (100 μm), **(E,E’)** (100 μm), **(F,F’)** (75 μm), **(G–H’)** (100 μm), and **(J–K’)** (0.2 mm).

The glycoprotein Reelin labels a major subpopulation of GABAergic interneurons (Guidotti et al., 2000). At P0, WT Reelin+ interneurons are expressed in a high-anterior to low-posterior gradient at the interface between marginal zone (MZ) and ULs (**Figure 6A**). At this stage, WT Reelin+ interneurons display a morphology that is intermediate between tangential and radial (**Figure 6B**). In contrast, in the KO there are very few cells distributed in the CP, and the anterior to posterior gradient is rudimentary (**Figures 6A’,B’**). In both WT and KO, Reelin+ Cajal-Retzius cells in the MZ are unaffected. At P7, Reelin+ interneurons are mainly located in layer 5 of the CP in WT, whereas in the KO they are dispersed and reduced in number (**Figures 6C,C’**). By P21, interneuronal migration is complete; but I observe a 32% reduction in the total number of Reelin+ interneurons in the KO (**Figure 6D**, 0.68 ± 0.07, *p* = 0.0261^∗∗^, *N* = 4). These data highlight a delay in the migration of Reelin+ GABAergic interneurons in the KO, with a severe reduction in their number.

**FIGURE 7:**
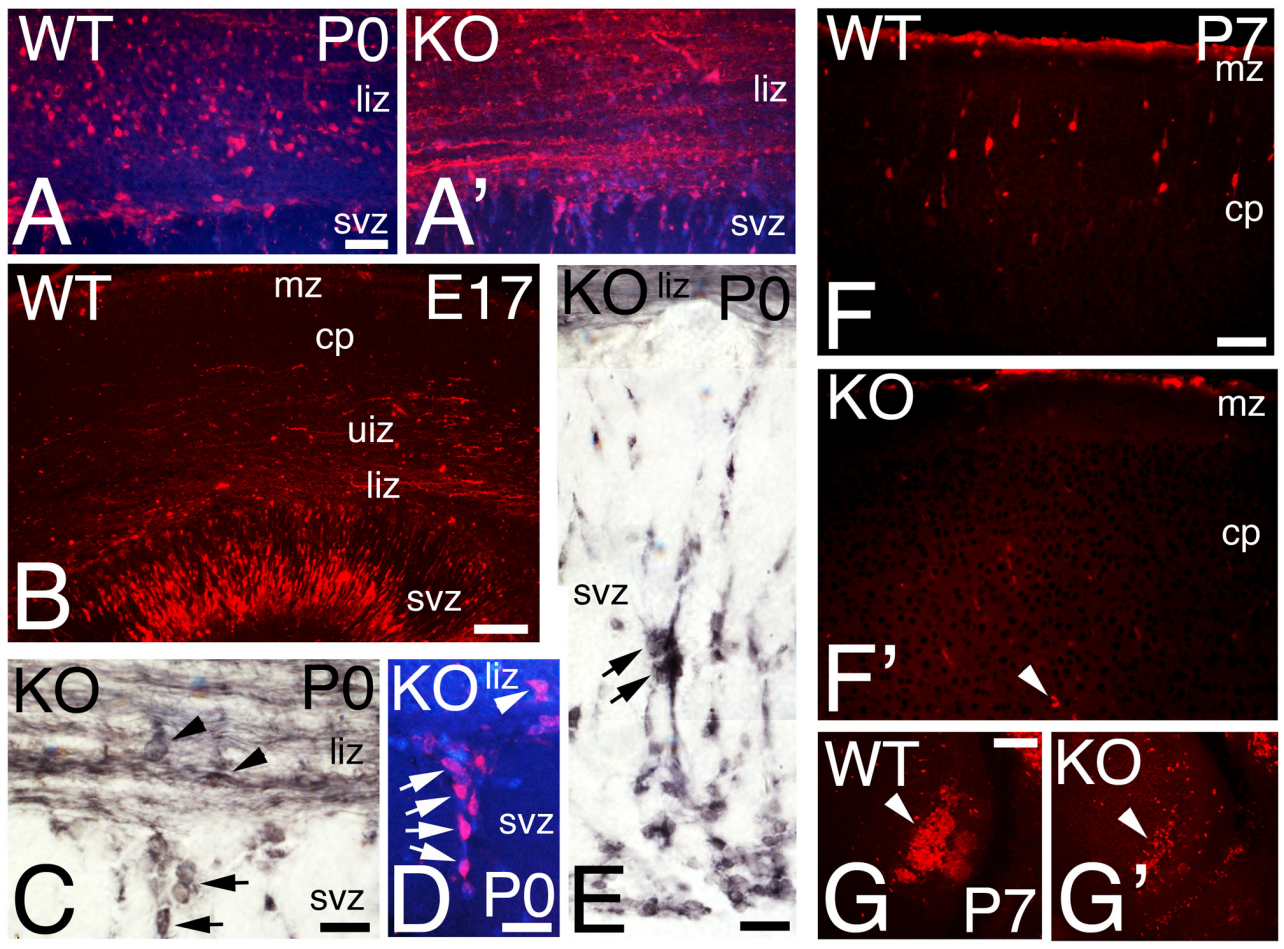
**Calretinin interneuronal subpopulation is deficient in the KO mice. (A–G)** P0 **(A,A’,C–E)**, E17 **(B)**, and P7 **(F–G’)** cortical sections of WT **(A,B,F,G)** and KO **(A,C–E, F’,G’)** labeled with Calretinin (CR). At P0, CR+ interneurons are radially oriented in the lower intermediate zone (liz) in WT **(A)** but they are tangentially oriented and embedded in a CR+ fiber plexus in the KO **(A’)**. At E17 WT, CR+ interneurons are radially oriented in the svz, **(B)**. **(C–E)** P0 KO, high power views showing in detail tangentially oriented CR+ interneurons immersed in the CR+ fiber plexus in the lower intermediate zone (arrowheads, **C**), with radially oriented interneurons migrating within the SVZ (arrows in **C–E**). DAPI is shown as counterstaining. **(F–G’)** At P7, CR+ interneurons are located in their final disposition in the cortical plate (cp) of WT **(F)** but they are almost absent in the KO (**F’**, arrowhead). High magnification of a caudal group of CR+ interneurons population severely reduced in the KO compare to WT (**G,G’**, arrowhead). mz, marginal zone, uiz, upper intermediate zone. Scale bars **(A,A’)** (50 μm), **(B)** (125 μm), **(C–E)** (25 μm), **(F,F’)** (75 μm), and **(G,G’)** (100 μm).

The calcium binding protein Calretinin (CR) is commonly used to label a subpopulation of GABAergic interneurons. CR+ interneurons migrate tangentially to the cortex using the interphase between intermediate zone (IZ) and SVZ (**Figures 6E,F**). At P0, WT CR+ interneurons are located in the lower IZ with a radial morphology, an indication consistent with migration to their final location in the CP (**Figures 6E,F** and **7A**). In the KO, in contrast to WT, CR+ interneurons located in the lower IZ are tangentially oriented and intermingled in a dense plexus of CR+ fibers (**Figure 7A’**). Concomitantly, radially oriented CR+ interneurons are located in SVZ (**Figures 6E’,F’** and **7C–E**), resembling E17 WT (**Figure 7B**). By P7, CR+ interneurons in the KO are nearly absent (**Figures 6G’** and **7F’**), whereas many CR+ interneurons are already positioned in the upper CP in WT (**Figures 6G** and **7F**). At this stage, a dense subset of CR+ interneurons is observed caudally in WT, but the same group is depleted in KO (**Figures 7G,G’**). By P21, the population of CR+ interneurons in the KO is very diminished compared to WT (**Figures 6H,H’**), with a very significant reduction by 35% in the total number of CR+ interneurons in the KO versus WT (**Figure 6I**, 0.66 ± 0.07, *p* = 0.0004^∗∗∗^, *N* = 9). As with Reelin, my data indicates a delayed migration of CR+ interneurons with a severe reduction in their number.

**FIGURE 8:**
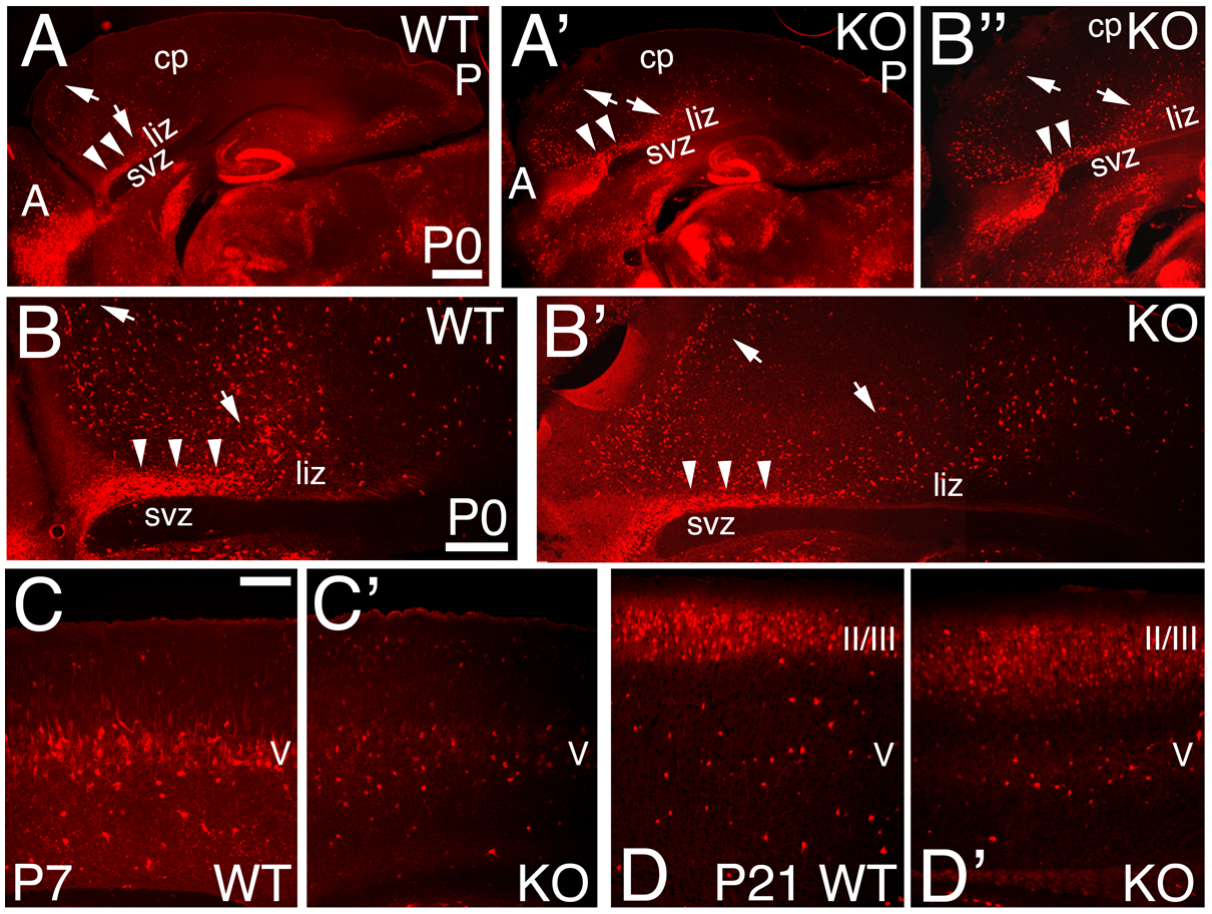
**Expression pattern of Calbindin positive interneurons in the KO mice. (A–D’)** Sagittal sections at P0 **(A–B”)**, P7 **(C,C’)**, and P21 **(D,D’)** in WT **(A,B,C,D)** and KO **(A’,B’,B”,C’,D’)** labeled with Calbindin (CB). At P0, CB+ interneurons reach the cortex using the interphase (arrowheads) between the lower intermediate zone (liz) and svz; from which they migrate radially to their final cortical position (arrows) without entering the SVZ. This pattern is preserved in the KO. **(B,B”)** are high magnifications of **(A,A’)**, respectively. By P7, CB+ interneurons are mostly located in layer V in WT, but they are severely reduced in the KO. At P21 WT, CB+ interneurons are densely packed in layers II/III and spread throughout the deep layers. In KO, CB+ interneurons are spread throughout layers II/III and localized in layer V. cp, cortical plate; II/III and V, cortical layers 2/3 and 5. Scale bars, **(A,A’)** (0.2 mm), **(B,B’)** (500 μm), **(B”)** (0.2 mm) and **(C–D’)** (100 μm).

The calcium binding protein Calbindin (CB) also labels a subpopulation of GABAergic interneurons. As observed at P0, CB+ interneurons tangentially migrate to the cortex using the lower IZ/SVZ interface (**Figures 8A,B**), but avoid entering the SVZ, and instead migrate radially to the CP following the anterior-to-posterior gradient (**Figures 8A–B”**). At P7, WT CB+ interneurons are densely located in layer 5; on the contrary, they are reduced in number in the KO (**Figures 6J,J’** and **8C,C’**). By P21, quantification shows a non-significant difference in the overall number of CB+ interneurons (**Figure 6L**; 1.12 ± 0.01, *p* = 0.186, *N* = 8). However, in the KO, their distribution pattern is altered with respect to WT, mostly in deeper layers (**Figures 6K,K’** and **8D,D’**).

**FIGURE 9:**
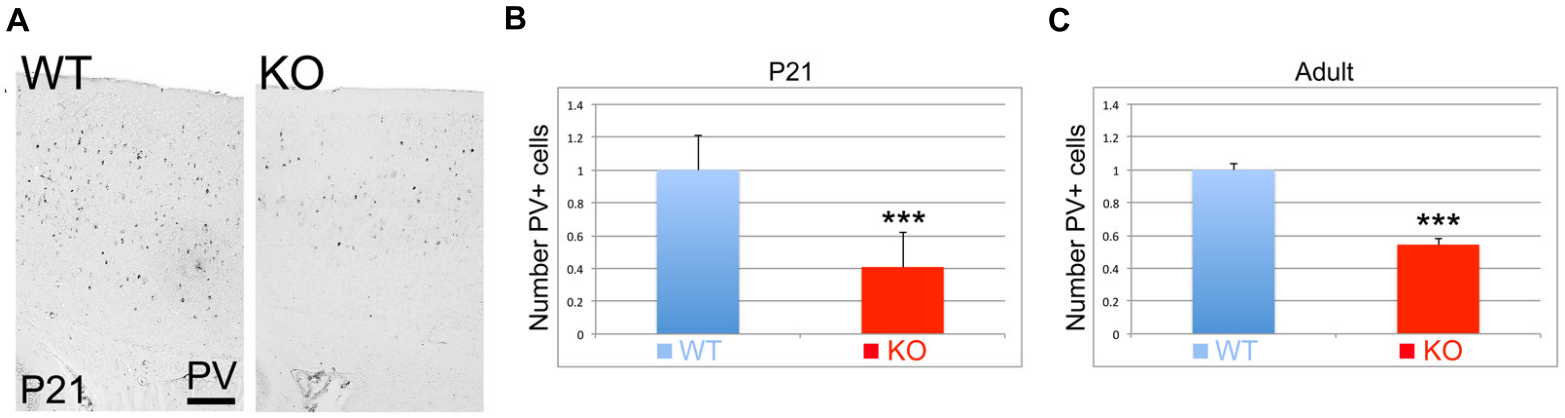
**Parvalbumin positive interneurons are reduced in the KO mice. (A)** Cortical sections immunostained for Parvalbumin (PV) at P21 show a reduction in the number of PV+ interneurons. **(B,C)** Quantitation analysis at P21 **(B)** and adult **(C)**. PV+ interneurons in the KO are significantly reduced by 59% at P21 (0.41 ± 0.07, *p* = 0.0001^∗∗∗^, *N* = 12) and by 46% in the adult (0.54 ± 0.08, *p* = 0.0001^∗∗∗^, *N* = 6). Scale bar: 100 μm.

Parvalbumin (PV) is considered a mature interneuronal marker expressed in almost half of the GABAergic interneurons in the adult cortex. At P21, I observe a reduction in the number of PV+ interneurons in the cortex of the KO versus WT (**Figure 9A**). Quantification analysis indicates that this reduction is very significant by 59% at P21 (**Figure 9B**, 0.41 ± 0.07, *p* = 0.0001^∗∗∗^, *N* = 12), and by 46% in the adult (**Figure 9C**, 0.54 ± 0.01, *p* = 0.0001^∗∗∗^, *N* = 6).

My data highlight deficits in migration, number, and distribution in major subpopulations of GABAergic interneurons in the KO, which is consistent with a role of the NRG-ErbB4 signaling pathway in interneuronal migration (Flames et al., 2004; Li et al., 2012).

### The Development of Specific Cerebellar Cell Types is ErbB4 Dependent

The cerebellum is a primary coordinator of motor function but recent evidence suggests that it also plays a key role in cognition (Leiner et al., 1991; Kim et al., 1994; Andreasen et al., 1999; Seidler et al., 2002; Andreasen and Pierson, 2008). To establish a correlation between ErbB4 and the development of the cerebellum, I analyze the main cell types involved in cerebellar function in the KO.

**FIGURE 10:**
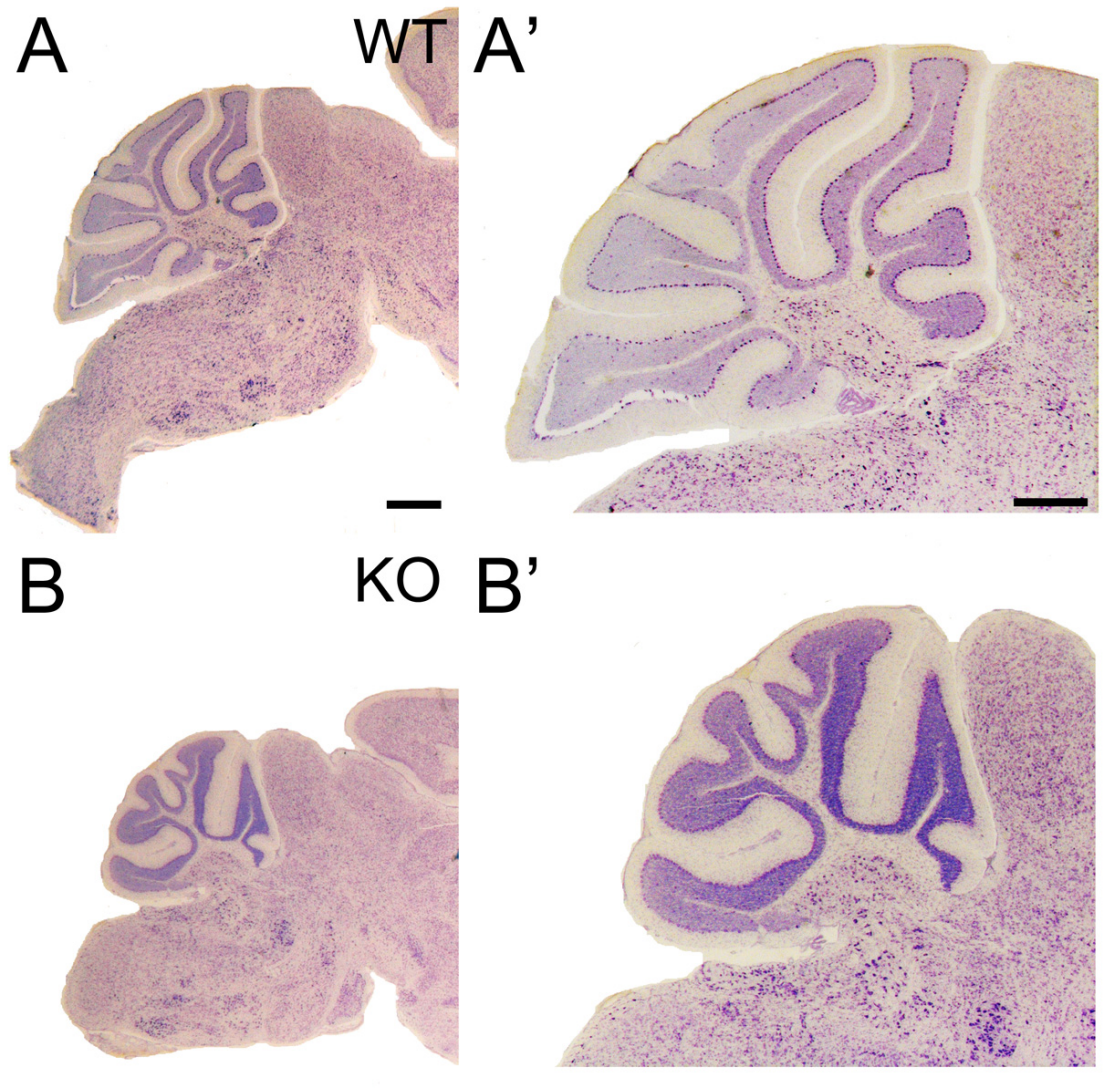
**Cerebellar volume is reduced and gyri foliation is poorly developed in the KO. (A–B’)** P21 cerebellar sections of WT **(A,A’)** and KO **(B,B’)** stained with Nissl. **(A’,B’)** are high magnifications of **(A,B)**, respectively. The volume of the cerebellum is reduced in KO compared to WT. It is noticeable the poorly developed gyri in some of the cerebellar lobes. Scale bars **(A,B)** (0.2 mm) and **(A’,B’)** (500 μm).

At P21, the development of the cerebellum is completed; however, a reduction in cerebellar size is observed in the KO, with poorly developed gyri in the cerebellar lobes (**Figures 10** and **2A**).

**FIGURE 11:**
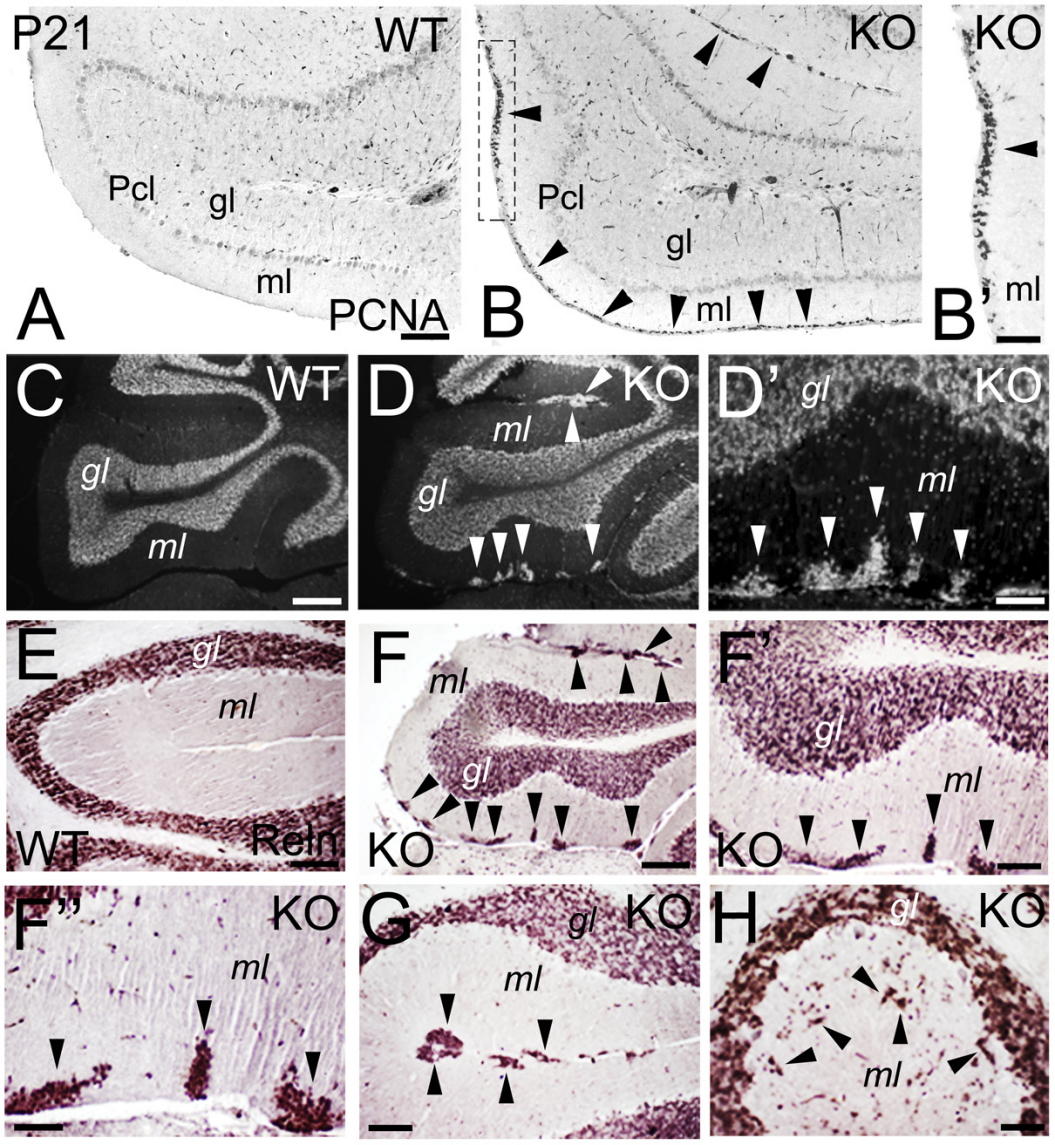
**Deficits in the migration and maturation of cerebellar excitatory granule cells suggest an ErbB4 dependent role. (A–B’)** WT **(A)** and KO **(B,B’)** cerebellar sections at P21. A stream of proliferative PCNA+ granule cells (arrowheads) is observed in the molecular layer (ml) of the KO. In WT, no PCNA+ proliferation is observed because the cerebellum is already mature. **(B’)** high power view of **(B)**. **(C–H)** WT **(C,E)** and KO **(D,D’, F–H)** cerebellar sections in adult mice. **(C–D’)** DAPI sections converted into monochromatic images showing granule cells in the granular layer (gl) of both WT and KO, in addition to ectopic granule cells in the molecular layer in the KO (arrowheads). **(D’)** high power view of **(D)**. **(E–H)** Reelin (Reln)+ granule cells in WT **(E)** and KO **(F–H)** labeling the granular layer of both WT and KO and the ectopic granule cells observed only in the KO (arrowheads). **(F’,F”)** are high magnifications of **(F)**. Pcl, Purkinje cell layer. Scale bars: **(A,B)** (100 μm), **(B’)** (50 μm), **(C,D)** (200 μm), **(D’)** (25 μm), **(E)** (50 μm), **(F)** (150 μm), **(F’)** (50 μm), **(F”)** (25 μm), and **(G,H)** (50 μm).

During cerebellar development, granule cells proliferate while migrating tangentially through the external granular layer (molecular layer in adult), and then use Bergmann glia to migrate radially into the internal granular layer (granular layer in adult). At P21, WT granule cells are located in the granular layer (**Figures 11A,C,E**). However, an abnormal proliferation, as marked by expression of the Proliferating Cell Nuclear Antigen (PCNA), is observed in the molecular layer of the KO, suggesting defects in the proliferation and migration of the granule cells (**Figures 11B,B’**). In the adult, granule cells are located in the granular layer in WT (**Figure 11C**). In the KO, the cell density of the granular layer is reduced and, surprisingly, ectopic cell clusters are observed in the molecular layer (**Figures 11D,D’**). To determine if these ectopic cells are indeed granule cells, I use Reelin as a marker. Reelin expression is located in the granular layer in WT (**Figure 11E**) and KO (**Figures 11F–H**); however, in the KO it also labels the ectopic clusters in the molecular layer (**Figures 11F–H**), indicating that these ectopic cells are neuronally differentiated and are, indeed, mature granule cells that are ectopically located (**Figures 11F–H**). These Reelin+ clusters in the molecular layer are primarily located tangentially (**Figures 11F,G**), but are also present radially (**Figure 11H**); which suggests an arrest in the migration of specific subpopulations of granule cells that, nevertheless, differentiate with their counterparts in the granular layer.

**FIGURE 12:**
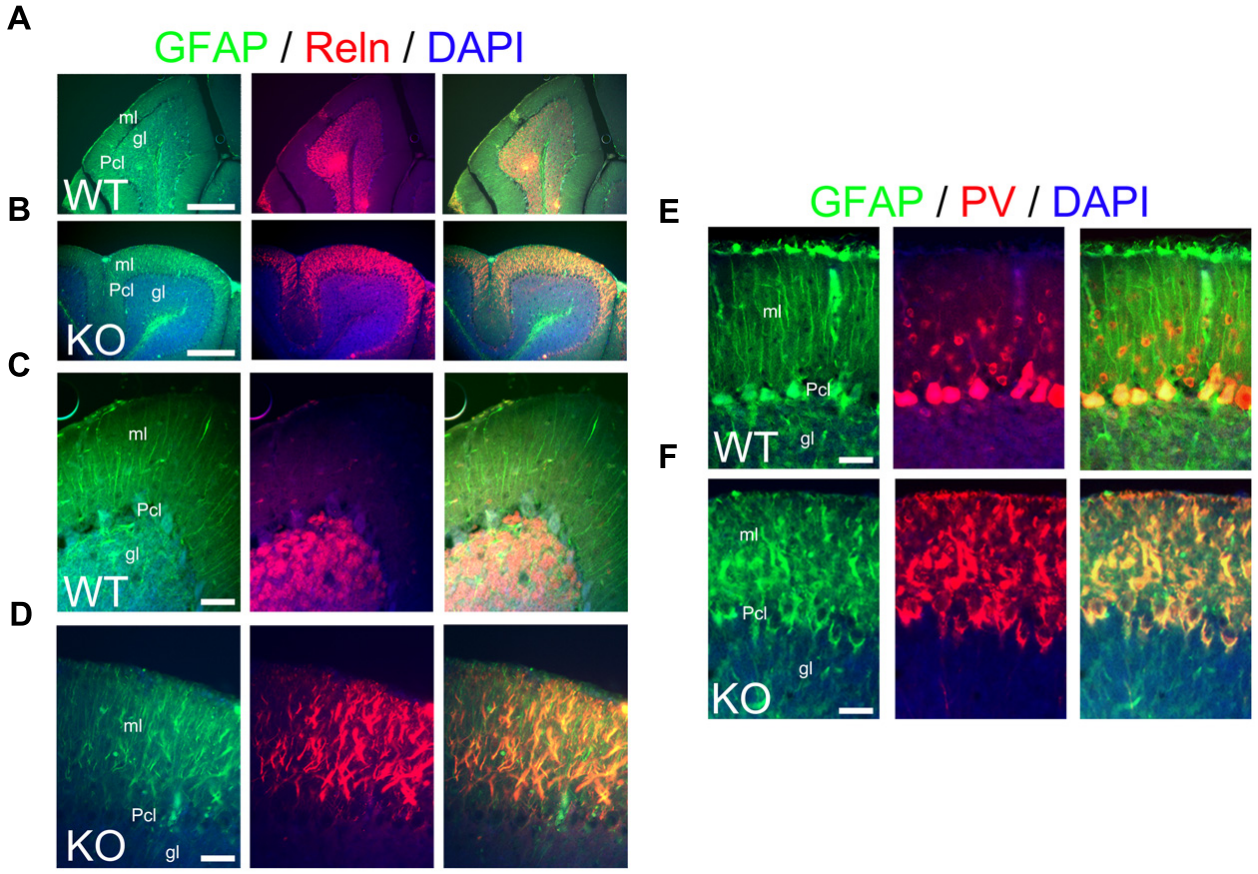
**The main inhibitory cell populations in the cerebellum are deficient in KO. (A–F)** WT **(A,D,E)** and KO **(B,D,F)** cerebellar sections at P21. **(A–D)** In WT, GFAP+ Bergmann glia cells (green) are embedded within the Purkinje cell layer (Pcl) and extend their radial fibers throughout the molecular layer (ml). Reelin (Reln, red)+ granule cells are located in the granular layer (gl). In KO, GFAP labels misaligned and fragmented radial fibers of the Bergmann glia with Reln+ granule cells embedded in an abnormal distribution within the molecular layer. DAPI is shown as counterstaining. Merge images are shown. **(C,D)** are high magnification panels of **(A,B)**, respectively. **(E,F)** As mentioned in **(A–D)**, GFAP labels normal and fragmented Bergmann glia in WT and KO, respectively. Parvalbumin (PV)+ interneurons are distributed throughout the molecular layer in WT but their distribution pattern is disrupted in the KO being intermingled in the misaligned Bergmann glia. PV also labels mature Purkinje cells, normally labeled in WT and with deficiencies in KO. Scale bars: **(A,B)** (250 μm) and **(C–F)** (50 μm).

Bergmann glia are a specialized type of cerebellar astroglia that express ErbB4 and are critical for granule cell migration and synaptic pruning (Rio et al., 1997). To define the status of Bergmann glia and their relation to granule cells in the KO, I use GFAP and Reelin as markers for Bergmann glia and granule cells, respectively. At P21, Bergmann glia in WT elaborate perfect GFAP+ radial fibers throughout the molecular layer with Reelin+ granule cells located in the granular layer (**Figures 12A,C**). In contrast, the KO presents fragmented and misaligned GFAP+ radial fibers with abnormal Reelin positivity in the molecular layer (**Figures 12B,D**).

Parvalbumin is commonly used to label cerebellar interneurons in the molecular layer where it also labels Purkinje cells (Leto et al., 2006). In WT at P21, PV+ interneurons are intermingled in the molecular layer, where Purkinje cells are also PV+ (**Figure 12E**). In the KO, PV+ interneurons are also intermingled in the molecular layer but their distribution and expression pattern is aberrant within the fragmented Bergmann glia (**Figure 12F**). This is consistent with the previously described abnormalities observed in the molecular layer.

**FIGURE 13:**
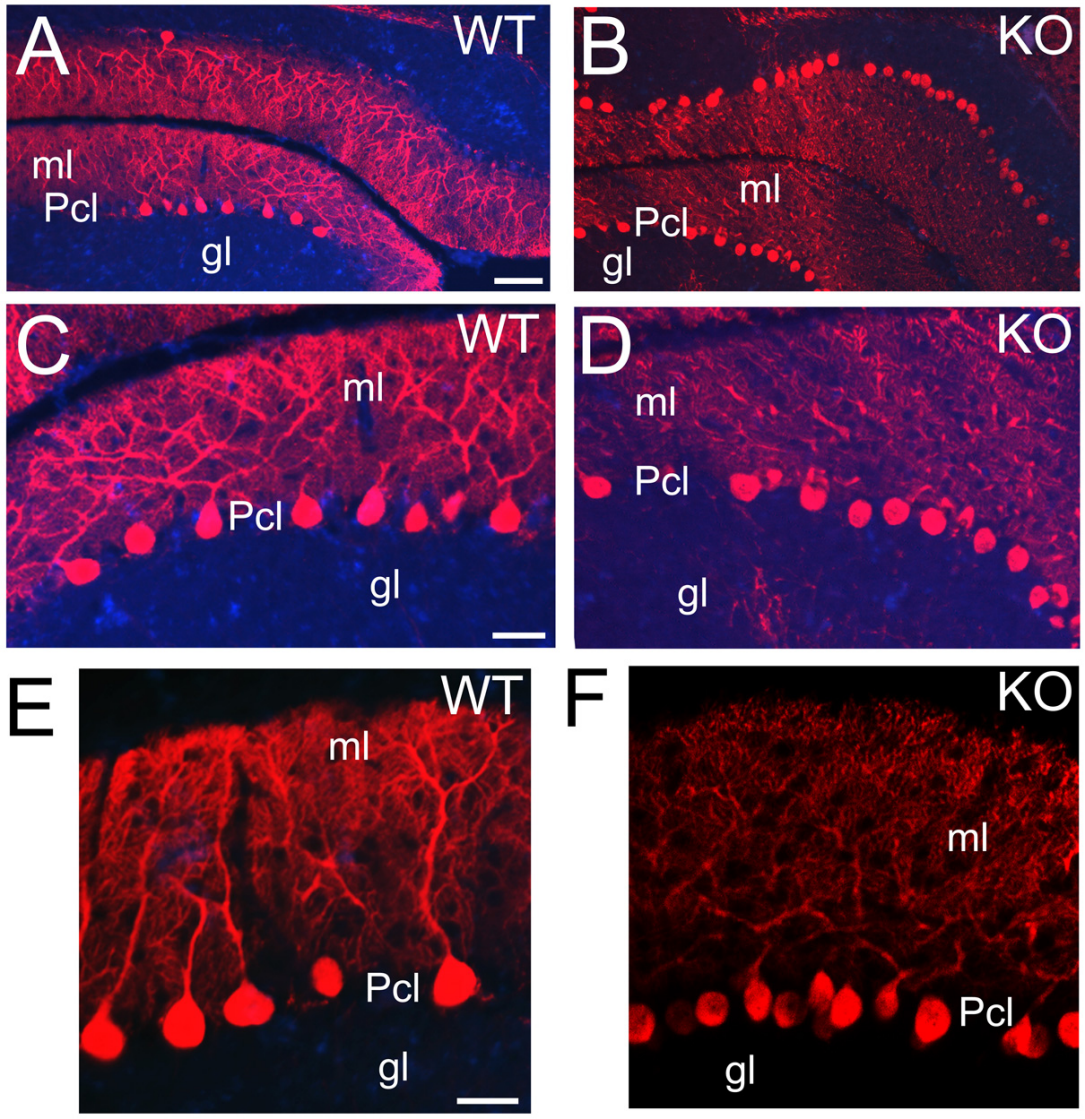
**ErbB4 is required during Purkinje cells development. (A,B)** WT **(A,C,E)** and KO **(B,D,F)** sections of cerebellum labeled with Calbindin (CB) at P21. CB+ Purkinje cells are distributed in a monolayer with their cellular bodies in the Pcl and their arborized dendritic trees embedded in the molecular layer (ml). This pattern is preserved in both WT and KO. However, in KO the Purkinje cells are reduced in size and in number and their dendritic trees are poorly developed. gl, granular layer. Scale bars: **(A,B)** (100 μm), **(C,D)** (50 μm) and **(E,F)** (25 μm).

Purkinje cells are characterized by their distribution in a monolayer with very elaborated dendritic arbors and large number of dendritic spines in the molecular layer (Leto et al., 2006). Purkinje cells are GABAergic and express calcium-binding proteins such as PV (**Figures 12E,F**) or CB (**Figure 13**). At P21, CB+ Purkinje cells are distributed in a monolayer with their arborized dendritic tree embedded in the molecular layer (**Figures 13A,C,E**). In the KO, Purkinje cells are also located in a monolayer but their cell bodies are smaller and their dendritic trees are underdeveloped with deficiencies in their arborization (**Figures 13B,D,F**).

**FIGURE 14:**
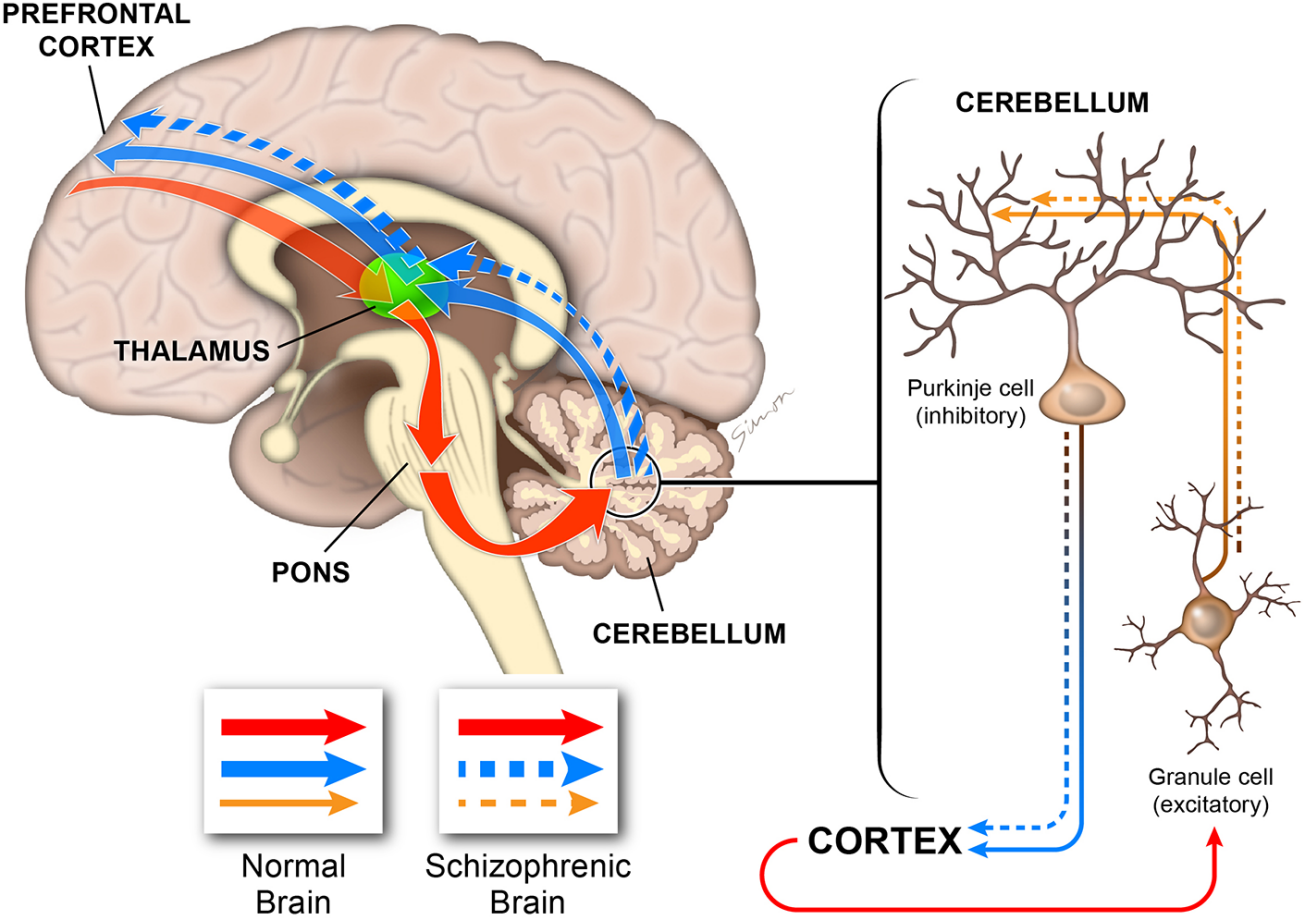
**Diagram illustrating the role of the cerebellum in cognitive processing.** The cerebellum is connected to multiple cortical regions by the cortico-cerebellar-thalamic-cortical circuit (CCTCC). As part of the CCTCC, the cerebellum performs an error detection task and, indeed, functions as a modulator of the cognitive information received from the cortex **(red)**. The inhibitory Purkinje cells and the excitatory granule cells play critical functions in maintaining the excitatory/inhibitory balance **(brown)** in the cerebellum, which is necessary to coordinate the cortical information received by the cerebellum to be able to generate a correct output back to the cortex **(blue)**. In schizophrenia, the cerebellum is not able to perform normally by filtering and coordinating the cortical information received **(brown dashed)** and, therefore, it generates a flawed output **(dashed blue)**. In this model, deficiencies during the development of the different cerebellar cell types, particularly Purkinje and granule cells, will alter the excitatory/inhibitory balance that is critical for cerebellar-dependent cognitive activity. Therefore, the cerebellar output will have errors with downstream consequences for cortical cognitive function as observed in schizophrenia.

My results strongly implicate the NRG-ErbB4 signaling pathway in cerebellar function and highlight severe defects in the main cell types involved in the development and functionality of excitatory/inhibitory systems in the cerebellum. The flaws in Purkinje and granule cells are consistent with deficiencies in the excitatory input to the Purkinje cells and an imbalance in the inhibitory output (**Figure 14**). Thus, the ability of the cerebellum to integrate cognitive cortical information and create an appropriate output signal back to the cerebral cortex is impaired (**Figure 14**), which is consistent with some of the cognitive impairments observed in schizophrenia (Tran et al., 1998; Andreasen et al., 1999; Levitt et al., 1999; Eastwood et al., 2001; Andreasen and Pierson, 2008).

### Hippocampal Maturation is Compromised in the KO

**FIGURE 15:**
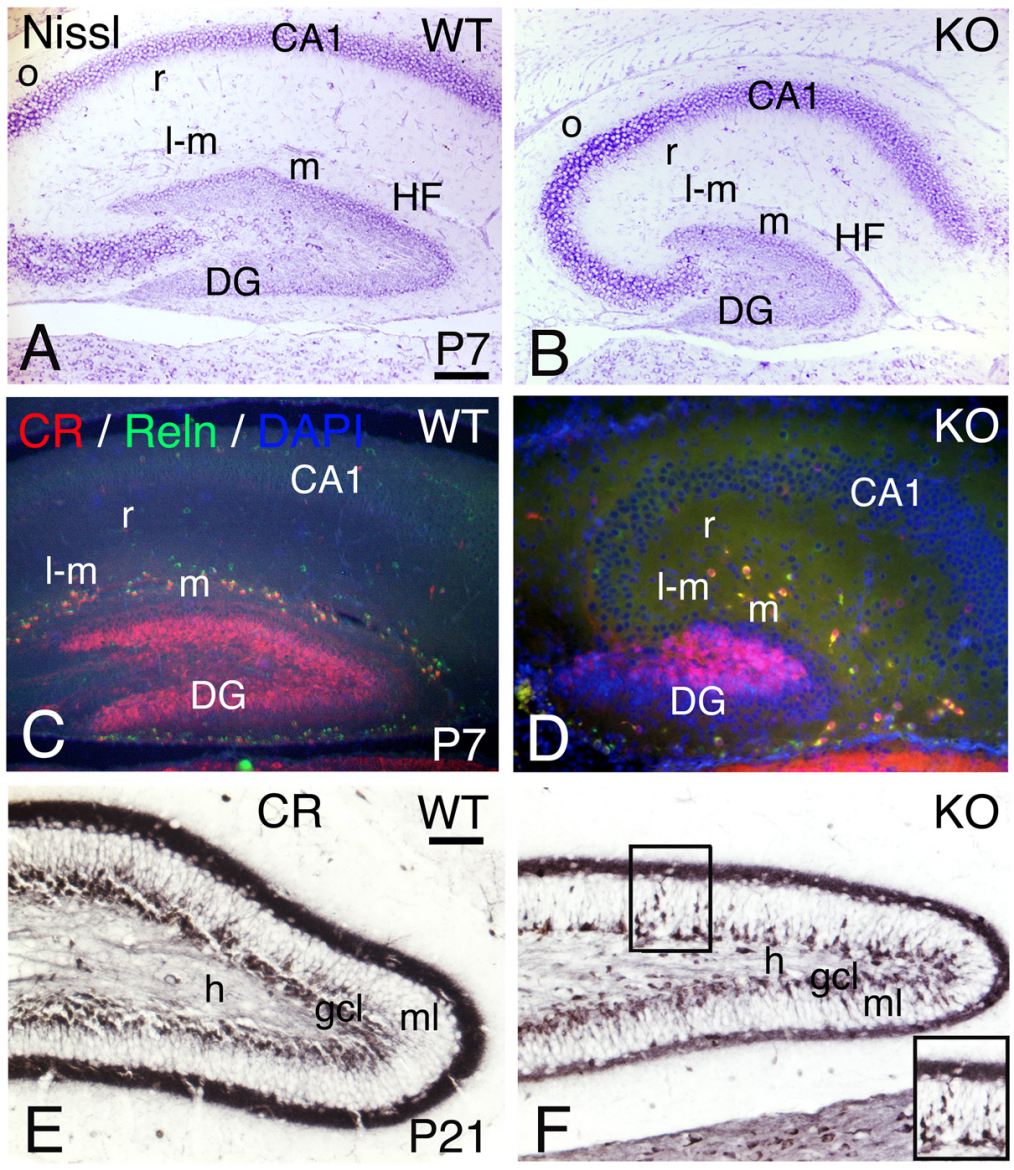
**Hippocampal development is compromised in the KO mice. (A,D)** (P7) and **(E,F)** (P21) hippocampal sections of WT **(A,C,E)** and KO **(B,D,F)**. **(A,B)** P7 Nissl staining shows a poorly developed hippocampus in the KO with a very rudimentary dentate gyrus (DG) and Cornus Ammonis (CA) fields. **(C,D)** At P7, the Calretinin (CR) and Reelin (Reln) interneuronal populations in the hippocampal strata lacunosum-moleculare (l-m), moleculare (m) and radiatum (r) are decreased in the KO compared to WT. **(E,F)** At P21, using Calretinin as a marker, I observe a fully developed dentate gyrus in WT. However, in the KO both dentate gyrus blades are thinner with a persistent migration of CR+ immature neuronal progenitors in the molecular layer (ml, inset in **F**). gcl, granular cell layer; h, hilus; HF, hippocampal fissure; o, strata oriens. Scale bars **(A–D)** (250 m) and **(E,F)** (125 μm).

Adult hippocampal interneurons express ErbB4 and their number is reduced in the ErbB4 KO mice (Neddens and Buonanno, 2010). To determine if the development of the hippocampus is altered in the KO model, I compared P7 and P21 (**Figure 15**).

A reduction in hippocampal volume with a poorly developed dentate gyrus is observed in the KO with respect to WT (**Figures 15A,B**). The interneuronal markers CR and Reelin are decreased in the stratum moleculare, stratum lacunosum moleculare, and stratum radiatum (**Figures 15C,D**), which is consistent with (1) previous reports using different interneuronal markers (Vullhorst et al., 2009; Neddens and Buonanno, 2010) and (2) similar defects observed in the cortex and cerebellum (present results). At P21, the KO dentate gyrus presents an undergoing cell migration of CR+ immature neuronal progenitors in the granular cell layer that is not observed in WT, which is consistent with a reduction in cellular density of both dentate gyrus blades (**Figures 15E,F**).

## Discussion

Brain development is a very complex process involving interconnected signaling pathways. Many of the genes in these pathways have reciprocal roles in different brain structures; therefore, insults to the pathways have collateral consequences in several neuroanatomical structures, affecting the integrity, connectivity and functionality of the adult brain. One of these pathways is the NRG1-ErbB signaling. NRG1 functions are largely mediated by a class of receptor tyrosine kinases (ErbB1-4) (Yarden and Sliwkowski, 2001; Falls, 2003), and through their activation regulate: radial glia formation and survival (Adlkofer and Lai, 2000), oligodendrocyte formation and axon myelination (Canoll et al., 1996; Fernandez et al., 2000; Schmucker et al., 2003), axon pathfinding (Lopez-Bendito et al., 2006), neuronal migration (Anton et al., 1997; Rio et al., 1997; Flames et al., 2004; Li et al., 2012), expression of neurotransmitter receptors (Ozaki et al., 1997; Rieff et al., 1999; Liu et al., 2001) and dendritic development (Rieff and Corfas, 2006).

### Neurodevelopmental Implications of the ErbB4 Deletion

My model of study is the *ErbB4^-/-^ HER^heart^* mice (referred as KO) (Tidcombe et al., 2003). This mouse model has been widely used for functional and behavioral studies, but a detailed neuroanatomical description of the KO defects has not been sufficiently analyzed. It has been previously reported an absence of gross-morphological defects in the KO at P21 (Flames et al., 2004). However, the detailed neuroanatomical description of this study differs from that assumption and, indeed, offers clear evidence that the cortical thickness in the KO is reduced at P7 by 25% (rostral: 0.75 ± 0.061, *p* = 0.0113^∗∗^; caudal: 0.74 ± 0.009, *p* = 0.0001^∗∗∗^) and in the adult by 6% (rostral: 0.94 ± 0.010, *p* = 0.0041^∗∗∗^; caudal: 0.94 ± 0.011, *p* = 0.0101^∗∗^), with a reduction in brain volume in the adult KO by 15%. (0.85 ± 0.02, *p* = 0.0027^∗∗∗^). This is consistent with: (1) defects in the migration and maturation of UL neurons, (2) reduced number of cortical GABAergic interneurons, (3) reduction in cell populations such as astrocytes and (4) similar defects observed in the hippocampus and cerebellum that also show a reduction in volume. The large amount of neurodevelopmental insults observed in the KO will likely contribute to the overall reduction in thickness and brain volume. Future directions will dissect out the mechanism of action between NRG1-ErbB4 signaling and the lamination in ULs.

The neuron-astroglia interactions are critical for the development and function of the nervous system (Haydon and Carmignoto, 2006; Oberheim et al., 2006). ErbB4 is expressed in GFAP+ astroglial progenitors and ependymal cells in cortical SVZ (present results, (Ghashghaei et al., 2006)). When glial ErbB receptors are blocked, NRG-expressing cells fail to induce radial glia formation, and the migration along the radial glial fibers is impaired (Anton et al., 1997; Rio et al., 1997). This is consistent with my observations in the cortex (GFAP astroglia) and in the cerebellum (Bergmann glia), where ErbB4-deficient glial cells cannot respond to the NRG-signaling, affecting their development and, subsequently, altering the migratory paths along the radial fibers. In the cortex, this deficiency will induce a premature transformation into astrocytes, and a significant reduction in their number. In the cerebellum, it will likely affect the migration of the granule cells as observed in the KO model. In the ErbB1 knockout mice, the astrocytes undergo excessive apoptosis (Wagner et al., 2006), suggesting that ErbB1 also plays a significant function in astrocyte development.

Oligodengrocytes are fundamental in the nervous system to provide support and insulation to axons by creating the myelin sheath (Nave, 2010). NRGs activation of ErbB2, ErbB3, and ErbB4 is essential for oligodendrocyte development and production of myelin (Riethmacher et al., 1997; Garratt et al., 2000; Chen et al., 2006; Roy et al., 2007; Brinkmann et al., 2008). The expression of a dominant-negative ErbB4 receptor (DN-ErbB4) in oligodendrocytes completely blocks ErbB2, ErbB3 and ErbB4 receptor signaling; thus, reducing the number of oligodendrocytes, inducing a delay in the onset of myelination, thinner myelin, reduction in the expression levels of myelin genes such as MBP, and slower conduction velocity in CNS axons (Roy et al., 2007). The hypomyelination observed using a DN-ErbB4 (Chen et al., 2006; Roy et al., 2007); or after conditional deletion of ErbB2 in glial cells (Garratt et al., 2000); or in mice with one copy of NRG1 (Michailov et al., 2004), is consistent with my data in the KO showing a reduction of MBP+ oligodendrocytes and cortical hypomyelination, more significant in ULs, which is also consistent with a maturation defect observed in UL neurons. Concurrent with the reduction in myelin, I also observe a failure of axonal development in the cortex that agrees with the reported roles of the NRG1-ErbB4 signaling pathway mediating axonal growth (Lopez-Bendito et al., 2006).

Cortical GABAergic interneurons are mostly generated in the Medial Ganglionic Eminence where they express ErbB4 (Yau et al., 2003). ErbB4-expressing interneurons migrate to the cortex through NRG1-induced chemotropic cues (Flames et al., 2004; Li et al., 2012). Deficiencies in this signaling pathway have been shown to affect the number of GABAergic interneurons in the cortex (Flames et al., 2004; Li et al., 2012). My findings in the KO are consistent with the reported reductions in number of cortical GABAergic interneurons. It is likely that these ErbB4-deficient interneurons are not able to properly respond to the NRG1 chemotropic cues, affecting their migration and final laminar distribution in the cortex.

The cerebellum is the primary coordinator of motor movement, and it has been recently implicated in higher order processes such as cognition (Leiner et al., 1991; Kim et al., 1994; Andreasen et al., 1999; Seidler et al., 2002; Andreasen and Pierson, 2008). The migration of granule cells influences cerebellar lamination and thereby cerebellar function. In the developing cerebellum, granule cells express NRG and Bergmann glia express ErbB4, and this interaction is necessary to induce the development of radial fibers by Bergmann glia (Rio et al., 1997). Conditional deletion of ErbB3 using a GFAP-Cre driver showed a reduction in cerebellar volume and an aberrant lamination in the cerebellum, with the presence of ectopic clusters of mature granule cells in adjacent cerebellar folia and a severe reduction and disorganization of the Bergmann glia scaffold (Sathyamurthy et al., 2015). This report shows similar results to mine using the ErbB4 KO model, where (1) mature granule cells fail to reach the internal granule layer, but instead form ectopic clusters in the molecular layer, indicating defects in the migration of granule cells; and (2) the Bergmann glia is severely affected and disorganized. The similarities between both phenotypes and the expression of ErbB3 and ErbB4, but not ErbB2, in Bergmann glia cells (Rio et al., 1997; Sathyamurthy et al., 2015) suggests that a heterodimer ErbB3/ErbB4 is likely the functional receptor activated by the NRG-expressing granule cells.

It is possible that additional defects specific to the migration and proliferation of granule cells occur in the KO, since a delayed and still active proliferation is present at stages when cerebellar development is completed. Granule cells are required for the normal dendritic differentiation of Purkinje cells (Altman and Bayer, 1997); furthermore, Bergmann glial cells, which cell bodies are located around Purkinje cells, are also involved in the growth and development of the Purkinje cells (Leto et al., 2006). Therefore, it is likely that the reduced dendritic tree observed in the Purkinje cells in the KO is due to a combinatorial effect of a defective development of both granule cells and Bergmann glia.

It has been previously reported that the population of interneurons in the hippocampus of the KO is reduced (Neddens and Buonanno, 2010), which is consistent with my observations using the same mouse model. However, I also observe defects in the maturation of the dentate gyrus, characterized by increased numbers of CR+ immature neuronal progenitors and thinner blades, phenomenon that has been described in human schizophrenia and bipolar patients (Walton et al., 2012). This is consistent with ErbB4 being expressed in the subgranular zone of the dentate gyrus (Neddens and Buonanno, 2010) and it is the first report that establish a correlation between ErbB4 and the development of the dentate gyrus with the pathophysiological alteration termed as “immature dentate gyrus” observed in schizophrenia (Walton et al., 2012).

### NRG-ErbB4 Signaling and Schizophrenia

Strong evidence suggests that schizophrenia is caused by cumulative defects during brain development that will ignite the disease in the adult before any clinical symptom is observed (Harrison and Weinberger, 2005; Walsh et al., 2008; Bale et al., 2010; Meyer-Lindenberg, 2010; Mitchell, 2011). Unfortunately, it is not understood what developmental insults define schizophrenia, and there is a gap between neurodevelopmental causes that determine the disorder and the onset of the first symptoms. Human genetic mapping and single risk variant studies of schizophrenic families have unveiled many gene candidates such as DISK-1, NRG1, ErbB4, or COMT (Stefansson et al., 2002, 2003; Law et al., 2007; Walsh et al., 2008; Meyer-Lindenberg, 2010). The majority of these genes play critical roles in brain development, such as the NRG1-ErbB4 signaling pathway that is involved in glia cell differentiation, myelination, neuronal migration, axon guidance, synapse formation, and synaptic plasticity; all deficient in schizophrenia (Mei and Xiong, 2008). A genetic disruption of ErbB4 has also been observed in schizophrenic patients (Walsh et al., 2008).

The ErbB4 KO model presents a complete array of neurodevelopmental defects consistent with the prognosis and development of the schizophrenia in the adult brain. Neuroimaging studies in schizophrenic patients indicate an overall reduction in brain volume (Volz et al., 2000; Shenton et al., 2001; Meyer-Lindenberg, 2010) suggesting defects during brain development that coincide with the reductions I observe in the ErbB4 model. Defects in neuronal migration have been reported in the disease, in particular the presence of heterotopias (Kovelman and Scheibel, 1984; Shenton et al., 2001; Muraki and Tanigaki, 2015), which is also consistent with my observations in the ErbB4 model.

Post-mortem studies in schizophrenic brains have shown deficits in astrocytes, oligodendrocytes, and in myelination (Flynn et al., 2003; Hof et al., 2003; Harrison and Weinberger, 2005; Catts et al., 2014). A significant decrease in the number of astrocytes has been observed in schizophrenic patients (Rajkowska et al., 2002; Webster et al., 2005), which is consistent with the reduction in the number of astrocytes I observe. My results also suggest an ErbB4-dependent role in oligodendrocytes development and myelin production, which coincide with previous studies in mouse models and schizophrenia (Canoll et al., 1996; Flynn et al., 2003; Hof et al., 2003; Roy et al., 2007).

Defects in the GABAergic system are a common hallmark in schizophrenia (Guidotti et al., 2000; Beasley et al., 2002; Hof et al., 2003; Hashimoto et al., 2008) and are highly recapitulated in mouse models (Flames et al., 2004; Li et al., 2012; Steinecke et al., 2012; Muraki and Tanigaki, 2015). Consistent with my data where specific subpopulations of GABAergic interneurons are more susceptible to deficits in ErbB4, reports in schizophrenic prefrontal cortex and hippocampus show specific reductions in Reelin, PV, and Somatostatin (Beasley et al., 2002; Hof et al., 2003; Harrison, 2004; Hashimoto et al., 2008). The contribution of specific subpopulations of GABAergic interneurons altering the GABAergic system in the brain might influence the progression of the disease. For example, PV-GABAergic interneurons are essential for the generation and oscillation of local gamma activity that is necessary for brain activity, plasticity, and connectivity (Buzsaki and Draguhn, 2004; Sohal et al., 2009). However, the contribution of other subpopulation of interneurons to the etiology of the disease is yet unknown.

Neuroimaging studies have revealed important roles for the cerebellum in cognition, and there is strong evidence of its involvement in schizophrenia (Andreasen et al., 1999; Andreasen and Pierson, 2008). The cerebellum connects with many cortical areas through the cortico-cerebellar-thalamic-cortical circuit (CCTCC) (Middleton and Strick, 1994, 2001; Andreasen et al., 1999; Andreasen and Pierson, 2008). Strong evidence suggests that disruptions in this cortical-subcortical-cerebellar circuitry or “cognitive dysmetria” might explain the impairments in motor and cognitive coordination associated with schizophrenia (Seidler et al., 2002; Mei and Xiong, 2008). Recent studies have shown a decrease in Purkinje cells size and their dendritic arborization, and a decreased excitatory input from granule cells due to a reduction in synaptic proteins (Eastwood et al., 2001). The balance between excitatory inputs from granule cells to inhibitory Purkinje cells is of course critical for cerebellar function and the coordination of cerebellar-dependent cognitive activity. The ErbB4 mouse is a good model to study cerebellar defects associated with schizophrenia, in which an imbalance in the inhibitory Purkinje cells/excitatory granule cells takes place, altering cognitive processing in the cerebellum with downstream consequences for cortical cognitive function, a characteristic symptom of schizophrenic patients (Andreasen et al., 1999; Andreasen and Pierson, 2008).

Neuregulin-1 and ErbB4 mouse models have been extensively studied for functional and behavioral studies since they show schizophrenia-related behaviors (Stefansson et al., 2002; Golub et al., 2004; O’Tuathaigh et al., 2007) including hyperactivity in the open field, defects in the Prepulse Inhibition (PPI) and deficits in fear conditioning and extinction (Stefansson et al., 2002; Golub et al., 2004; O’Tuathaigh et al., 2007; Chen et al., 2008, 2010; Mei and Xiong, 2008; Shamir et al., 2012). Some of these behavioral defects such as the fear conditioning can be reverse using antipsychotic drugs such as clozapine (Mei and Xiong, 2008; Barros et al., 2009). The heterozygous reeler mice also develop schizophrenia-related behaviors such as deficits in PPI (Barr et al., 2008). My findings indicate a significant reduction in Reelin+ interneurons in the ErbB4 KO, which is consistent with the reported reductions in Reelin observed in schizophrenic patients (Fatemi et al., 2000; Guidotti et al., 2000). Therefore, some interaction between the NRG1-ErbB4 signaling and other schizophrenia-related genes such as Reelin might occur during development.

This study offers a detailed neuroanatomical description of the neurodevelopmental defects associated to the ErbB4 deficiency, which is a valuable resource to establish a direct cause-effect correlation with the functional and behavioral studies observed in schizophrenia-related mouse models, in particular those involving the NRG1-ErbB4 signaling pathway.

Schizophrenia is not caused by a single gene malfunction, but rather by a cascade of cumulative neurodevelopmental deficiencies associated with many genes and pathways. Nonetheless, perturbation of NRG-ErbB4 signaling provides a comprehensive biological model that appears to impinge upon many of these pathways. Indeed, this model unveils new possibilities with respect to our understanding of the biological basis of the disease.

## Author Contributions

CGP-G designed the study, prepared the experiments, analyzed findings, prepared the figures, and wrote the paper.

## Conflict of Interest Statement

The author declares that the research was conducted in the absence of any commercial or financial relationships that could be construed as a potential conflict of interest.
